# UBA52 Is Crucial in HSP90 Ubiquitylation and Neurodegenerative Signaling during Early Phase of Parkinson’s Disease

**DOI:** 10.3390/cells11233770

**Published:** 2022-11-25

**Authors:** Shubhangini Tiwari, Abhishek Singh, Parul Gupta, Sarika Singh

**Affiliations:** 1 Division of Toxicology and Experimental Medicine, CSIR-Central Drug Research Institute, Lucknow 226031, India; 2 Academy of Scientific & Innovative Research (AcSIR), Ghaziabad 201002, India

**Keywords:** Parkinson’s disease (PD), protein aggregation, endoplasmic reticulum stress, ubiquitin-proteasome system (UPS), ubiquitin-60S ribosomal protein L40 (UBA52), dopaminergic neuronal death

## Abstract

Protein aggregation is one of the major pathological events in age-related Parkinson’s disease (PD) pathology, predominantly regulated by the ubiquitin–proteasome system (UPS). UPS essentially requires core component ubiquitin; however, its role in PD pathology is obscure. This study aimed to investigate the role of ubiquitin-encoding genes in sporadic PD pathology. Both cellular and rat models of PD as well as SNCA C57BL/6J-Tg (Th-SNCA*A30P*A53T)39 Eric/J transgenic mice showed a decreased abundance of UBA52 in conjunction with significant downregulation of tyrosine hydroxylase (TH) and neuronal death. In silico predictions, mass spectrometric analysis, and co-immunoprecipitation findings suggested the protein–protein interaction of UBA52 with α-synuclein, HSP90 and E3-ubiquitin ligase CHIP, and its co-localization with α-synuclein in the mitochondrion. Next, in vitro ubiquitylation assay indicated an imperative requirement of the lysine-63 residue of UBA52 in CHIP-mediated HSP90 ubiquitylation. Myc-UBA52 expressed neurons inhibited alteration in PD-specific markers such as α-synuclein and TH protein along with increased proteasome activity in diseased conditions. Furthermore, Myc-UBA52 expression inhibited the altered protein abundance of HSP90 and its various client proteins, HSP75 (homolog of HSP90 in mitochondrion) and ER stress-related markers during early PD. Taken together, the data highlights the critical role of UBA52 in HSP90 ubiquitylation in parallel to its potential contribution to the modulation of various disease-related neurodegenerative signaling targets during the early phase of PD pathology.

## 1. Introduction

The age-related movement disorder Parkinson’s disease (PD) is caused due to the loss of dopaminergic neurons of the substantia nigra in the midbrain [1,2,3] The neuropathological hallmarks of the disease include Lewy bodies containing protein (α-synuclein, ubiquitin, chaperones) aggregates, reflecting the impaired protein degradation mechanisms such as ubiquitin-proteasome system (UPS) and autophagy [2]. Pertained organelle endoplasmic reticulum (ER) dysfunction has been reported in PD by us and others ([4,5,6,7,8,9,10] that eventually leads to dopaminergic neuronal death. In concurrence, we recently showed the cardinal role of eukaryotic initiation factor 2α during the early phase of PD pathology [11]. The dysfunctional ER physiology leads to the accumulation of aberrant and unassembled proteins that need to be degraded by proteasome machinery/UPS. Reduction in proteasome activity during PD has also further indicated the critical role of UPS in disease pathogenesis ([12,13]. UPS assertively requires the attachment of ubiquitin moiety to the target protein for their degradation with the mandatory requirement of enzymes E1 (ubiquitin-activating enzyme), E2 (ubiquitin conjugation protein) and E3 (ubiquitin ligase), a process called ubiquitylation. Tagging of ubiquitin on the substrate proteins, through lysine residues, either leads to their degradation through proteasome or asserts their participation in various intracellular pathways ([2]. Since ubiquitin moiety is an indispensable requirement of UPS, this study is focused on ubiquitin-encoding genes during the early phase of PD pathology. Ubiquitin is a 76 amino acid-containing moiety encoded by the four genes- UBB, UBC, UBA52 and RPS27a. UBB and UBC encode for the polyubiquitin chains, whereas UBA52 and RPS27a are fusion proteins with ubiquitin residue on the N-terminus and ribosomal protein (L40 and S27a) on the C-terminus. In addition, the key participation of the related regulatory ubiquitin ligases has also been evaluated.

The role of UBA52 has been reported in other pathologies; however, we are the first to report its critical role in PD pathology. In traumatic brain injury, altered mRNA and protein levels of UBA52 have been observed [14], while in diabetic nephropathy and hepatoma cell apoptosis, upregulated UBA52 was found [15]. In embryonic development, the role of UBA52 has also been reported as UBA52 deficient mice exhibited a decrease in protein synthesis, cell cycle arrest and death [15].

The RPL40 subunit of UBA52 participates in the initiation, translation and elongation process through its interaction with the eukaryotic elongation factor-2 (eEF2) [16]. Emerging shreds of evidence suggest that in PD, the phosphorylation of eEF2 takes place, which promotes its dissociation from the ribosome and stalls the mRNA translation process, thereby stimulating the α-synuclein aggregation and consequent dopaminergic cytotoxicity [17]. Although unreported, UBA52 and upregulated phosphorylated levels of eEF2 might have neurodegenerative implications during PD pathology that need to be studied and, henceforth, oblige us to first understand the role of ubiquitin genes in PD pathology. Along with this prime objective, the study was further extended to understand the implication of UBA52 in chaperone functioning and biochemical alterations, specifically related to the mitochondrion and ER organelle during PD pathology.

The ER organelle is primarily responsible for folding the nascent polypeptide with the help of chaperones and targets the misfolded proteins toward the UPS. Since we have already depicted the insinuation of ER signaling in PD, in this study, we evaluated the role of the involved chaperone in regulating protein machinery. HSP90 is one of the known predominant chaperones that co-localizes with α-synuclein in Lewy bodies during α-synucleinopathies and is associated with impaired proteasome function [18]. The activity of HSP90 is also modified by the co-chaperone HSC70. Burmann et al. 2020 [19] previously reported that the selective inhibition of α-synuclein interaction with HSC70 as well as with HSP90, especially with HSP90β, leads to its re-localization in the mitochondrion and subsequent formation of protein aggregates. It has been reported that during cellular stress, such aberrant and misfolded proteins are recognized by molecular chaperones, which assist the E3 ligases, such as the C-terminal of Hsp70 interacting protein (CHIP), Parkin, Siah1/2 and others, in their ubiquitylation and degradation by the UPS [20]. E3 ligase, CHIP interacts with chaperones HSP70 and HSP90 to participate in the protein quality control (PQC) and UPS-mediated degradation of HSP90 and α-synuclein, a component of Lewy bodies [21]. In addition to CHIP, another E3 ligase Parkin also interacts with and ubiquitylates α-synuclein-interacting protein, synphilin-1. The co-expression of synphilin-1, α-synuclein and Parkin leads to the formation of ubiquitin-positive Lewy body inclusions, suggesting a key role of Parkin in ubiquitylating proteins and participation in Lewy body formation [22]. Additionally, it has also been suggested that PINK1 phosphorylates both ubiquitin and Parkin to fully activate Parkin for its utmost E3 ligase activity, leading to its translocation to the damaged mitochondrion in order to regulate the pathogenic events [23], suggesting the close association of ubiquitin and E3 ligases in energy-dependent degenerative mechanisms.

In view of the conjectural role of UBA52 in neuronal viability, its interference in the protein translation, and PQC through chaperones, the present study has been focused on gaining insight into the versatile role of UBA52 during the early phase of sporadic PD, utilizing both cellular and experimental rat models.

## 2. Materials and Methods

### 2.1. Chemicals and Antibodies

Please refer to Supplementary Table S1.

### 2.2. Cell Culture, Differentiation and Treatments

The human neuroblastoma SH-SY5Y, mouse neuroblastoma N2a and rat pheochromocytoma PC12 cells were maintained in Dulbecco’s modified Eagle’s medium (DMEM) and F12 (1:1) media supplemented with penicillin/streptomycin and 10% fetal bovine serum (FBS) or 10% FBS and 5% horse serum for PC12 cells under the atmosphere of 37 °C and 5% CO_2_. Cells were cultured in culture flasks or culture well plates at 37 °C in a water-saturated atmosphere of 95% air and 5% CO_2_. Neurotoxin (rotenone) treatment was given in SH-SY5Y for 24 h to mimic the PD conditions [24,25]. For α-synuclein-PFFs (preformed fibrils)-induced experiments, 5 μg/mL of recombinant human α-synuclein protein PFF (Abcam, ab218819) was given in SH-SY5Y cells and then processed for immunofluorescence labeling and co-localization studies [26]. The basal proteasome activity of SH-SY5Y cells was inhibited with MG132 (cell-permeable proteasome inhibitor) treatment at a concentration of 5 μM for 6 h, prior to any other respective experimental set-up/treatment. In a set of experiments, the cells were treated with tunicamycin (ER stress inducer) at 1 µM for 24 h, as a positive control for ER stress. Experiments were also conducted in differentiated SH-SY5Y cells, N2a cells and PC12 cells, cultured in DMEM and F12 (1:1) media supplemented with penicillin/streptomycin and 1% FBS at 37 °C and 5% CO_2_. *All-trans* retinoic acid (ATRA, 10 μM) was added to the differentiating media of SH-SY5Y and N2a cells every alternate day for 7 days before assessing for morphological changes in differentiated cells [27,28], followed by rotenone treatment to SH-SY5Y (500 nM) and N2a (1 μM) for 24 h. For PC12 cells, 50 ng/mL of nerve growth factor (NGF) was added every alternate day for 7 days to induce differentiation and rotenone treatment (1 μM) was given for 24 h [10]. For chronic in vitro studies in SH-SY5Y cells, the cells were differentiated in differentiating media containing 10 μM of ATRA till the 7th day. Following differentiation (referred to as day 0), cells were exposed to 50 nM rotenone every alternate day till the 14th day and cells were then processed for immunofluorescence and immunoblotting [29]. All the experiments were repeated at a minimum of three times (represented as ‘n_exp_’ in figure legends) for statistical significance.

### 2.3. Plasmids, Cloning, Mutagenesis and Transfection

Myc-DDK tagged wild-type human UBA52 (Myc-UBA52) and Myc-DDK tagged wild type human α-synuclein (Myc-α-SYN) plasmid constructs were purchased from OriGene technologies, whereas UBA52^K48R^ (Flag-UBA52^K48R^), UBA52^K63R^ (Flag-UBA52^K63R^) mutants were customized and purchased from GenSript. All the plasmids were propagated in DH5α cells, followed by their isolation using a commercial plasmid isolation kit (Promega, Madison, Wisconsin, USA). For ectopic expression of the transients, the expression plasmids were transfected in SH-SY5Y cells using lipofectamine 3000 based on manufacturer instructions. The total RNA was extracted after 24 h of incubation [30]. The cDNA was obtained by RT-PCR and amplified through PCR using respective primers (OriGene Technologies, Rockville, MD, USA) followed by agarose gel electrophoresis to assess the gene expression. An empty vector, pcDNA3.1 was used where necessary in order to adjust the DNA amounts.

### 2.4. siRNA Mediated Knockdown

Pre-designed siRNA constructs were used for RNA interference, procured from Invitrogen (AM16708; Assay ID 102888) along with a scrambled siRNA (4390843). Transient silencing of UBA52 was performed using lipofectamine 3000, as described in the transfection kit (L3000-001).

### 2.5. Animals and Stereotactic Neurosurgery

The SNCA C57BL/6J-Tg (Th-SNCA*A30P*A53T) 39Eric/J transgenic mice (Stock number: 008239) were purchased from the Centre of Cellular and Molecular Biology (CCMB), Hyderabad, India and used in this study as a transgenic mice model (CSIR-CDRI (IAEC/2021/SI no2)) to assess the interaction of UBA52 with α-synuclein in genetically induced PD [31]. The genotyping of 12-month-old mice was performed using DNA extracted from their tail tips, followed by the PCR assay (cycling conditions: 94 °C for 5 min (94 °C for 15 s, 65 °C for 45 s and 72 °C for 30 s) * 35 and 5 min at 72 °C) obtained from the genotyping protocol database of Jackson Laboratories [32]. The primers used for genotyping are mentioned in Supplementary Table S2.

For sporadic neurotoxin-induced experiments, the outbred strain of male rat (Sprague–Dawley) weighing 200–220 g was procured from the National Laboratory Animal Centre of CSIR-Central Drug Research Institute, Lucknow, India. All conducted experiments followed the strict guidelines of the Institutional animal ethics committee (CSIR-CDRI (IAEC/2018/F-52)). Four animals were maintained in each polyacrylic cage with food and water ad libitum. Standard ambient conditions with a 12 h light and dark cycle, room temperature 22 ± 1 °C and humidity 60–65% were provided. The rats were divided into two groups with 4 animals per group. The groups were control (sham-operated DMSO-injected) and diseased (rotenone-administered sporadic PD). The experimental rats were anesthetized using a mixture of xylazine (10 mg/kg) and ketamine (80 mg/kg) and mounted on the stereotaxic apparatus (Stoelting, USA), and a specific region was located by measuring coordinates from bregma [33]. Unilateral administration (on the right side of the rat brain) of rotenone (dissolved in 3 μL DMSO, 6 µg in each region) was given in the substantia nigra (SN) and striatum (STR) region [11]. Appropriate care of rats was taken to prevent mortality, and after completion of the experimental duration (3 days post administration), the rats were sacrificed, and both SN and STR were isolated and processed for various assays or the brains were fixed for histological or immunofluorescence studies.

For α-synuclein-PFFs (preformed fibrils)-induced experiments, 4 μL of 2 μg/μL of recombinant mouse α-synuclein protein PFF (Abcam, ab246002) was unilaterally administered in the striatum region of the rat brain, and simultaneously, 4 μL of PBS was injected in the sham-operated control rats [34]. Appropriate care of rats was taken to prevent mortality, and after completion of the experimental duration (15-, 30- or 45-days post administration of α-synuclein protein PFF), the rats were anesthetized, followed by intra-cardiac perfusion with 0.9% saline and decapitated to remove the brain quickly [35]. SN and STR regions were isolated from the brain and the tissues were processed for various assays.

All the experiments have been repeated a minimum of three times (represented as ‘n_exp_’ in figure legends) for statistical significance.

### 2.6. Animal Behavior Assessment

*Neuromuscular coordination:* Rotarod assay was performed to assess the neuromuscular coordination in the control and rotenone-treated SD rats [36,37]. Briefly, the rats were trained for 3 consecutive days on the rotarod apparatus during a habituation trial of 5 min at 5–20 rpm to learn the motor balance. Before sacrifice, the rats were subjected to three rotarod sessions at 20 rpm for 2 min each. Data were manually recorded for all experimental groups, and the frequency of falls was calculated per minute.

*Stereotype behavior:* Apomorphine (1 mg/kg) was given to control and rotenone-administered SD rats by intraperitoneal route to assess the stereotypical behavior. After 30 min of apomorphine injection, the rotations were recorded on a live-video system. Rotations were observed for 2 min, and only full-body rotations were counted. Data were articulated as contralateral rotations per minute.

### 2.7. Cell Viability

Cell viability of SH-SY5Y was estimated using a mitochondrial dehydrogenase activity assay (tetrazolium dye-based colorimetric test), as reported previously [38]. The absorbance was read at 550 nm wavelength by a spectrophotometer (Gen5, BioTek, Santa Clara, CA, USA), and the mean of optical density was illustrated in graphs.

### 2.8. mRNA Expression by RT-PCR & qPCR

RT-PCR was performed as reported previously [30]. For qPCR, the PCR mixture was amplified in a DNA thermal cycler (Applied Biosystems, Waltham, MA, USA) for 35 cycles and the products were identified on a 2% agarose gel electrophoresis containing ethidium bromide. The respective primers were procured from Integrated DNA Technologies (Supplementary Table S2). Images were captured by a UVI gel documentation system and intensity was measured by Image J software.

### 2.9. Protein Extraction and Western Blot

The protein was extracted from the cell and tissue lysates (buffer composition: HEPES 200 nM pH-7.4, sucrose 250 mM, KCl 10 mM, MgCl2 1.5 mM, EGTA 1 mM pH-7.4, EDTA 1 mM pH-7.4, DTT 1 mM, PMSF, 1 mM, nonidet P40 0.05%, protease inhibitor cocktail and pepstatin A 1 mM), as reported previously, and immunoblotting was performed [39] using primary antibodies with appropriate secondary antibodies. The immunoblots were visualized using ChemiDoc XRS+ (Bio-Rad, Hercules, CA, USA) after developing the signal with substrate Femto Lucent plus HRP (G-Biosciences). The mean intensity (intensity/area) of bands was determined and normalized against β-actin using ImageJ software (NIH, USA).

### 2.10. Immunocytochemistry/Immunohistochemistry and Counterstaining

***Immunocytochemistry (ICC):*** The SH-SY5Y cells were seeded on the poly-l-lysine coated coverslips, and treatment was given for 24 h. ICC was performed in control, treated and transfected cells, as reported previously [30]. The signals were captured through fluorescent (Nikon eclipse E200, Japan) or confocal microscopy (Carl Zeiss, Jena, Germany).

***Immunohistochemistry (IHC):*** After the treatment, rats were sacrificed, and the brain was perfused with 15–20 mL of PBS. Quickly, each brain was isolated and kept in 4% sucrose solution for 3 h, incubated in 10% sucrose solution for another 3 h, and finally, shifted to 30% sucrose solution and kept overnight at 4 °C. The brains were then stored at −20 °C for 2 h, blocks were prepared with cryomatrix (Invitrogen, Waltham, MA, USA), and sections were cut down using a cryostat (Thermo Scientific, Waltham, MA, USA) to the thickness of 10–15 microns. The sections were collected on the poly-l-lysine coated slides immediately and processed for staining. Finally, the sections were mounted using an anti-fade medium containing counter stain DAPI (nuclear stain), and images were captured by fluorescent microscope (Nikon eclipse E200, Japan).

### 2.11. Thioflavin-S (Th-S) Assay

This assay was performed to assess protein fibril formation. The cells were seeded on a poly-l-lysine coated coverslip, followed by transient expression of Myc-UBA52 and rotenone treatment. Following treatment, the cells were washed in PBS and fixed with 4% PFA in PBS as mentioned above. For staining, the cells were incubated with Th-S stain (0.05% prepared in ethanol) for 30 min at room temperature in the dark. The cells were washed thrice with 70% ethanol, then rinsed once with PBS and mounted on slides using an anti-fade medium with DAPI to be visualized under a fluorescent microscope (Nikon eclipse E200, Japan).

### 2.12. Confocal Microscopy

Confocal imaging was performed in SH-SY5Y cells to visualize the localization/co-localization of α-synuclein, UBA52 and HSP90 [30]. After staining, the cells were mounted in DAPI containing an anti-fade mounting medium. The slides were visualized by a confocal microscope (Carl Zeiss, Jena, Germany), and images were captured in a single z-confocal axis.

### 2.13. Co-Immunoprecipitation (Co-IP)

SH-SY5Y cells or dissected rat brain tissue samples were lysed in a lysis buffer containing phosphatases and proteases. An equal amount of lysis extract (1–2 mg) was incubated with anti-UBA52 antibody and protein A-Sepharose beads to make a complex with end-over-end rotation overnight at 4 °C. Non-specific bound proteins were removed 4–5 times by washing with chilled PBS, and the immunocomplexes were boiled with 2x Laemmli buffer at 95 °C for 10 min to denature the existing proteins. The proteins were resolved on 4–20% gradient SDS-PAGE gel and either immunoblotted with respective antibodies for visualizing the proteins using chemiluminescence or processed to prepare the sample for mass spectrometric analysis.

### 2.14. Mass Spectrometry

Cell or tissue lysates were resolved on a 4–20% gradient pre-cast SDS-PAGE and later incubated in Coomassie blue (G-250) for 2–4 h till the gel was stained followed by de-staining (50:40:10:dH_2_0: Methanol: glacial acetic acid solution) at room temperature. Once the separated bands became visible, the de-staining solution was removed, and individual bands were sliced using a fine blade and immersed in microcentrifuge tubes (MCTs) containing the de-staining solution to remove the stain completely. Next, the gel slices were hydrated in 50–100 μL of 25 mM ammonium bicarbonate (ABC) solution (Step 1). This was followed by dehydrating the gel slices in 50 µL of solution A (2:1 mixture of acetonitrile: 50 mM ABC) for 5 min at room temperature (Step 2). The above steps were repeated till the gel slices became transparent. Next, the gel slices were vortexed for 5 min in 100% acetonitrile (ACN) and evaporated on a speed vacuum for 30 min at 45 °C for complete dehydration. The gel slices were rehydrated with 150 ng of trypsin (Sigma, Germany) and incubated on ice for 60 min. After the gel slices were completely rehydrated, 50–100 μL of 25 mM ABC solution was added, and gel slices were incubated overnight at 37 °C. The following day, the supernatant was taken in a new MCT, and each gel slice was dipped in 20–30 µL of 50% ACN and 2% trifluoroacetic acid (TFA) and vortexed for 5 min. All these extractions were added to the MCT containing the previous supernatant and evaporated on a speed vacuum till completely dry. The lyophilized sample was resuspended in 30% ACN and 0.1% TFA (5–10 µL) and again vortexed for 15 min to completely dissolve the sample. The volume of 0.75 μL of the sample was mixed with an equal amount of matrix (α-cyano-4-hydroxycinnamic acid (α-CHCA)) (1:1), spotted on a MALDI plate and processed using AB Sciex 4800 MALDI-TOF/TOF mass spectrometer. Positive ion spectra over *m/z* 800–4000 Da were recorded for analysis of UBA52 interacting proteins. From each spectrum, a maximum of twenty-five precursors with a minimum signal:noise ratio was carefully chosen for MS/MS analysis. A representative peptide spectrum against each sample was identified using Protein Pilot (AB Sciex, Framingham, MA, USA), and corresponding UBA52 interacting proteins were predicted using MASCOT and the NCBInr database.

### 2.15. In Vitro Ubiquitylation Assay

For in vitro ubiquitylation of HSP90 and α-synuclein, the cell and tissue lysates were prepared using lysis buffer. Co-immunoprecipitation was performed using anti-UBA52 antibody-protein A-Sepharose beads complex, and the immunocomplexes obtained were rinsed with chilled PBS 4–5 times and loaded on the vial, containing the reaction components provided in the in vitro ubiquitylation assay kit (Enzo life sciences, New York, NY, USA). The assay was performed based on the manufacturer’s instructions at 37 °C for 90 min, and the reaction was terminated after the addition of 2x non-reducing Laemmli buffer. The samples were resolved on SDS-PAGE, immunoblotted with anti-HSP90 and anti-α-synuclein antibodies, followed by their visualization using chemiluminescence.

### 2.16. Proteasome Activity

Proteasome activity was estimated in both cell (WT and Myc-UBA52) and tissue lysates as reported by us previously [40] utilizing trypsin (Z-ARR-AMC) and chymotrypsin (SUC-LLVY-AMC) substrate (Enzo life sciences, New York, NY, USA). Reaction mixtures were incubated for 60 min at 37 °C in the dark, and the fluorescence intensity was measured using a fluorimeter (Varian Cary Eclipse, USA) at an excitation/emission of 360/460 nm, respectively.

### 2.17. Statistical Analysis

Data were analyzed using Student’s unpaired t-test or one-way analysis of variance (ANOVA), and the difference between control and treated sets was analyzed by post hoc Dunnett’s multiple comparisons or Newman Keul’s test. The data generated upon assessing the effect of Myc-UBA52 in neurotoxin-induced studies were analyzed using two-way ANOVA, followed by Tukey’s-multiple comparison test. Values are expressed as the mean ± SEM, and a *p*-value less than 0.05 was considered statistically significant.

## 3. Results

### 3.1. Parkinson’s Disease-Specific Pathological Markers and UBA52

During the physiological condition, the cellular protein aggregates are degraded through protein degradation mechanisms such as UPS, which mandatorily requires ubiquitin to process target proteins for their degradation through proteasome machinery. With this hypothesis, we first performed the in silico analysis utilizing various database platforms such as BGEE (https://bgee.org/, accessed on 10 October 2022) and NCBI- Geo profile (https://www.ncbi.nlm.nih.gov/geoprofiles/, accessed on 10 October 2022) to check the available data of ubiquitin encoding genes in PD-related brain regions. The BGEE database suggested high basal expression of UBB, UBC, UBA52 and RSP27a genes in PD-related brain region-substantia nigra (SN). Next, we explored various publicly accessible microarray data available in the GEO profiles to observe the expression level of ubiquitin genes (UBB, UBC, UBA52, RPS27a) in the human brain (control and PD) as well as in rat brain (depending on the available database). We identified a GEO profile, GDS2821, showing the low abundance of UBA52 in the PD post-mortem brain in comparison to the healthy control human post-mortem brain. A microarray-based gene expression profiling performed in *post mortem* substantia nigra of the human brain of control and PD subjects showed a significant depletion of UBB (5.9 folds) and UBA52 (2.1 folds) in the PD brain [41]. However, the role of UBB in neurodegenerative disease is well studied [42,43,44], and therefore, we performed studies to check alterations in the gene expression and protein level of UBA52 during the onset of PD.

Based on the in silico inference and previous preliminary findings, we conducted the experiments to validate the alteration of UBA52 in PD-specific pathological markers in both sporadic cellular and rat experimental models and SNCA C57BL/6J-Tg (Th-SNCA*A30P*A53T) 39Eric/J transgenic mice.

Rotenone is a potent toxin that causes mitochondrial complex I inhibition, selectively leading to nigrostriatal dopaminergic neurodegeneration and motor deficits [45,46,47]. In contrast to 6-OHDA, it is also shown that the rotenone-injected SNpc region contains proteinaceous inclusions, such as Lewy bodies, immunoreactive for ubiquitin and alpha-synuclein [45].

**Figure 1:**
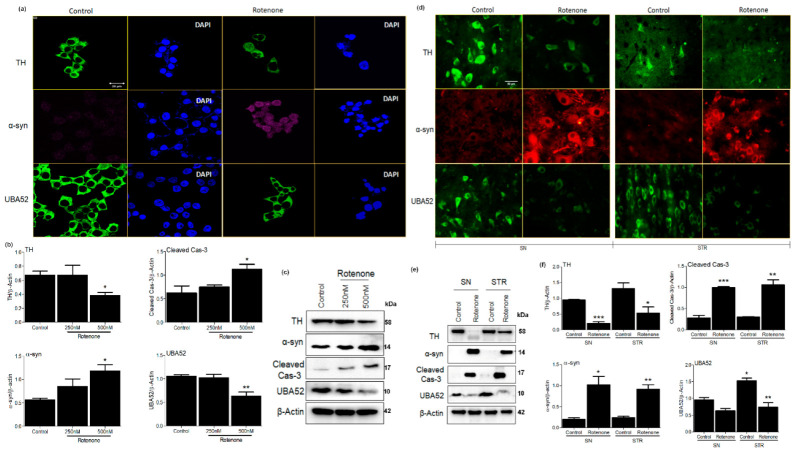
Parkinson’s disease markers and UBA52 expression in SHSY5Y and rat brain. (**a**) Confocal microscopy images depicting expression of TH, α-syn and UBA52 after secondary labeling with Alexa fluor -488 (green) and -546 (red), -647 (pink) in control vs. rotenone treated SH-SY5Y cells; n = 3. (**b**,**c**) A graphical and pictorial illustration of Western blot performed to assess the expression of TH, α-syn, cleaved cas-3 and UBA52, normalized against β-Actin in human SH-SY5Y neuroblastoma cells; n = 3. (**d**) Florescent images depicting the expression of TH, α-syn and UBA52 after secondary labeling with Alexa fluor -488 (green) and -546 (red) in control vs. rotenone treated SH-SY5Y cells, scale bar: 50μm; n = 3. (**e**) Immunoblot images illustrate the protein level of TH, α-syn, cleaved cas-3 and UBA52, normalized against β-Actin in rat brain regions. (**f**) Graphical representations of Western blot images normalized against β-Actin in SN and STR rat brain regions indicate a similar pattern as observed in SHSY5Y cells; n = 3. Quantifications are the mean and SEM of at least three independent experiments, and statistical analyses were performed using Student’s *t*-test and one-way ANOVA. * *p* < 0.05, ** *p* < 0.01, *** *p* < 0.001 control vs. Rotenone.

In line with this evidence, the selection was performed. First, the dose-ranging was performed to observe the appropriate concentration of rotenone that causes PD-related pathological conditions along with cell viability assessment in SH-SY5Y cells (Supplementary Figure S1a,b). A significant decrease in cell viability along with depletion in mRNA level of tyrosine hydroxylase (TH) was observed at both 250 and 500 nM doses of rotenone. Next, we evaluated the stability of PD pathology in the employed rotenone-induced experimental rats by estimating the mRNA levels of TH at different time points (till 21 days of rotenone administration) in both the SN and STR regions of the brain. Data suggested persistent depletion of TH in the experimental rat model till 21 days, which was initiated on the 3rd day after rotenone administration (Supplementary Figure S1c). Findings are in concordance with the study of Faull & Laverty (1969) and Bové et al. (2005) [48,49], which showed the maximal depletion of dopaminergic neurons at 3 days post neurotoxin administration. This might be due to the employed dose as well as the sites of injection (both SN and STR) of rotenone in the rat brain. We, therefore, selected the 3-day time point to study intracellular signaling mechanisms at the early/initiatory phase of the disease. The behavioral parameters (apomorphine-induced rotations and rota rod assay) also showed significant impairment in neuromuscular coordination after 3-day of disease induction (Supplementary Figure S1d,e). Concurrently, we utilized the same cDNA samples that were used to assess the TH mRNA to further check the gene expression of ubiquitin genes. Experiments confirmed the depletion in mRNA level of UBA52 in both substantia nigra (SN) and striatum (STR) regions of rat brain (Supplementary Figure S1f), whereas the levels of UBB, UBC and RPS27a were apparently not altered (Supplementary Figure S2). Simultaneously, we observed the low abundance of UBA52 mRNA in neuronal cell line SH-SY5Y after treatment (Supplementary Figure S1g). In line with the above findings, we further estimated the protein level of UBA52, TH, α-synuclein and cleaved caspase-3 in both SH-SY5Y cells and in SN and STR regions of rat brain. Data showed significant alteration in the protein level of UBA52, TH, α-synuclein and cleaved caspase-3 after rotenone administration in both experimental models (Figure 1a–f). However, in SH-SY5Y cells, the alteration in the protein level of TH and UBA52 was significantly profound at 500 nM concentration (Supplementary Figure S1); therefore, further experiments were conducted at a 500 nM concentration of rotenone.

### 3.2. UBA52 Surplus Attenuates the PD-Specific Pathological Markers and Neuronal Death

**Figure 2:**
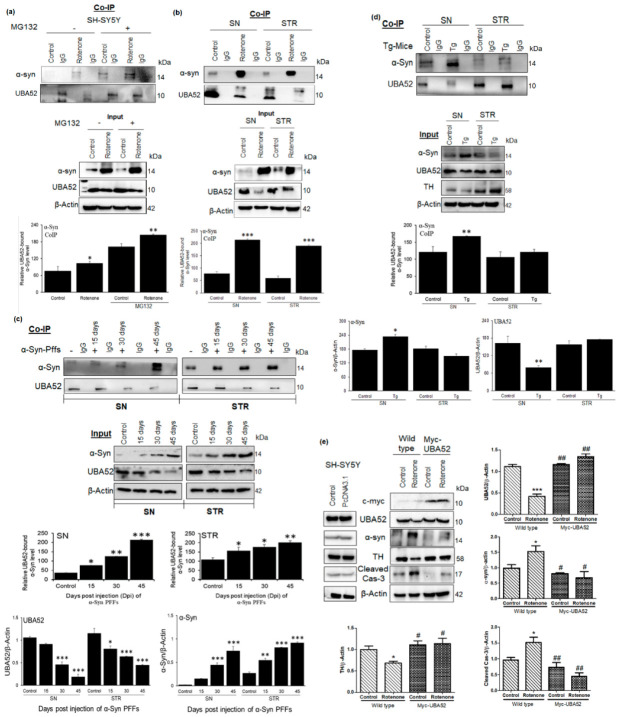
UBA52 interacts with α-Syn, and its over-expression in SH-SY5Y cells deter the alteration in PD markers protein abundance, preventing the disease onset. Co-IP (Protein A Sepharose beads attached to the anti-UBA52 antibody/anti-IgG antibody) was performed, and the cell or tissue lysates were incubated with the antibody-beads complex, following which the beads were boiled with 2X-reducing buffer, centrifuged and the supernatant was resolved on a 4–16% gradient SDS-PAGE to detect the interacting proteins. (**a**,**b**) Representative images and analysis depicting the in vitro interaction of UBA52 and α-syn, against IgG as a negative control in SH-SY5Y cells with or without rotenone& MG132 treatment; and control& rotenone treated PD-related SN and STR region, respectively; rats used per group in each replicate = 4; n_exp_ = 4. (**c**) Representative image and analysis depicting the Co-IP experiment to check the interaction of UBA52 and α-Syn, against IgG as a negative control in PBS-administered control& mouse recombinant α-Syn PFF treated PD-related SN and STR region, respectively at 15-, 30- and 45-days post-injection (dpi) to show the underlying mechanism of UBA52 in regulating α-Syn abundance; rats used per group per replicate = 5; n_exp_ = 3. (**d**) Representative image and analysis depicting the Co-IP experiment to check the interaction of UBA52 and α-Syn, against IgG as a negative control in the PD-related SN and STR region of the control C57BL mice and the C57BL/6J-Tg (Th-SNCA*A30P*A53T) 39Eric/J transgenic (Tg) mice, respectively; mice used per group per replicate = 5; n_exp_ = 2. (**e**) PcDNA3.1 or Myc-UBA52 transfected SH-SY5Y cells with or without rotenone treatment were immunoblotted for PD-related protein markers; n_exp_ = 3. Quantification is the mean and SEM of at least three independent experiments, and statistical analysis was performed using two-way ANOVA, followed by Tukey’s multiple comparison test. * *p* < 0.05, ** *p* < 0.01, *** *p* < 0.001 control vs. Rotenone/αSyn PFFs/Transgenic (Tg); # *p* < 0.05, ## *p* < 0.01.

Having determined that UBA52 was decreased in both in vitro and in vivo rotenone-treated experimental models, we then sought to validate the role of UBA52 in the alteration of disease-specific pathological markers. Primarily, in silico analysis indicated the interaction of UBA52 with TH and α-synuclein as assessed through a freely accessible server, PSOPIA (prediction server of protein-protein interaction) based on both human and rat protein sequences available on the UniProtKB database [50]. Results indicated that UBA52 interacts with α-synuclein (S_all_-0.9123) to a greater extent in comparison to TH (S_all_-0.3613) (Supplementary File S1). Further, to confirm the interaction of UBA52 with α-synuclein, we performed co-immunoprecipitation in both utilized rotenone-treated models. Observations validated in silico findings and showed that PD induction enhanced the interaction of UBA52 and α-synuclein. It might be because UBA52 is a ubiquitin gene, and it interacts with misfolded proteins or proteins with increased abundance during disease pathogenesis to enhance their proteasome-mediated degradation and restore homeostasis (Figure 2a,b).

α-synuclein preformed fibrils (PFFs) efficiently participate in seeding the aggregation and fibril formation of the endogenous α-synuclein. Therefore, to check the in vivo relevance or underlying mechanism for the direct UBA52-dependent regulation of α-synuclein via interaction, we performed the co-immunoprecipitation study in the SN and STR regions of recombinant mouse α-synuclein protein PFF-injected SD rats. Consistent with the hypothesis, we observed a time-dependent increase in the interaction of UBA52 and α-synuclein in the striatum (Figure 2c) along with the time-dependent altered protein levels of both UBA52 and α-synuclein (Input bands). However, in the SN region, the interaction was observed 30 days post-injection (dpi), which profoundly increased at 45 dpi (Figure 2c). The protein abundance of UBA52 was visibly reduced from 30 to 45 dpi, opposite to the change in the α-synuclein protein, which increased from 30 to 45 dpi (input bands).

Next, the interaction of UBA52 and α-synuclein was further assessed in the Tg-SNCA mice to confirm their interaction in the mutant α-synuclein model. Data obtained showed that this interaction was visibly higher in the SN region of the Tg-SNCA mice in comparison to the control C57BL mice (Figure 2d). The protein level of UBA52 was reduced in the Tg mice, whereas the α-synuclein protein level increased in the SN region of the mice. However, in the STR region, we did not observe a stark difference in the interaction of UBA52 and α-synuclein as well as in the protein abundance of UBA52 and α-synuclein (Figure 2d). It may be due to the middle age (12 months) of the transgenic mice, wherein the observed changes occurred in the SN region, and the old-aged mice (15–23 months) would be useful for further establishing the interaction profile of UBA52 and α-synuclein in the STR region of the brain. Parallelly, we also found only slight depletion in TH protein levels in the SN region with a rather increased TH abundance in the STR region, confirming the previous reports [51,52], which showed that α-synuclein mutation using transgenic approaches do not elicit the neurodegeneration in DA neurons.

Further, the SH-SY5Y cells were transiently overexpressed for UBA52, and we observed that UBA52 overexpressed cells exhibited significant attenuation of rotenone-induced upregulated α-synuclein and TH depletion along with inhibited neuronal death (cleaved caspase-3 level) (Figure 2e). UBA52 is a highly expressed ubiquitous gene (expression score-99.8 on BGEE database https://bgee.org/gene/ENSG00000221983/ (accessed on 10 October 2022), comparable to beta-actin with expression score-100), and its overexpression was apparently not visible through the direct UBA52 protein level but rather through c-Myc expression in transiently overexpressed cells. Altered protein folding and degradation during stress conditions is one of the most studied phenomena in PD pathology [2,37]; therefore, next, the protein fibril formation corresponding to aggregate generation was assessed through thioflavin staining. In agreement, UBA52 overexpressed cells showed inhibited fibril formation in comparison to wild-type cells during the early time-point of disease induction (Supplementary Figure S3a). Since α-synuclein is the major protein associated with PD and a prime component of the Lewy body along with chaperones and ubiquitin, we further assessed the level of α-synuclein by immunostaining. Rotenone treatment in the SH-SY5Y cells significantly exhibited the augmented level of α-synuclein, which was suppressed after Myc-UBA52 overexpression in SH-SY5Y cells (Supplementary Figure S3b). The implication of UBA52 transient overexpression was also confirmed in the recombinant human α-synuclein protein PFF-treated SH-SY5Y cells, and the immunofluorescent images suggested that UBA52 overexpression prevented the tendency of the human α-synuclein protein to form the toxic fibrils (Supplementary Figure S3c–d). These data revealed the in vitro and in vivo relevance for the direct UBA52-dependent regulation of α-synuclein via interaction, along with the strong implication of UBA52 in regulating the PD-specific pathological markers and protein aggregation tendency during the acute phase of disease pathogenesis.

### 3.3. UBA52 Revokes the PD-Specific Pathophysiological Markers in Differentiated Neuronal Cells

**Figure 3:**
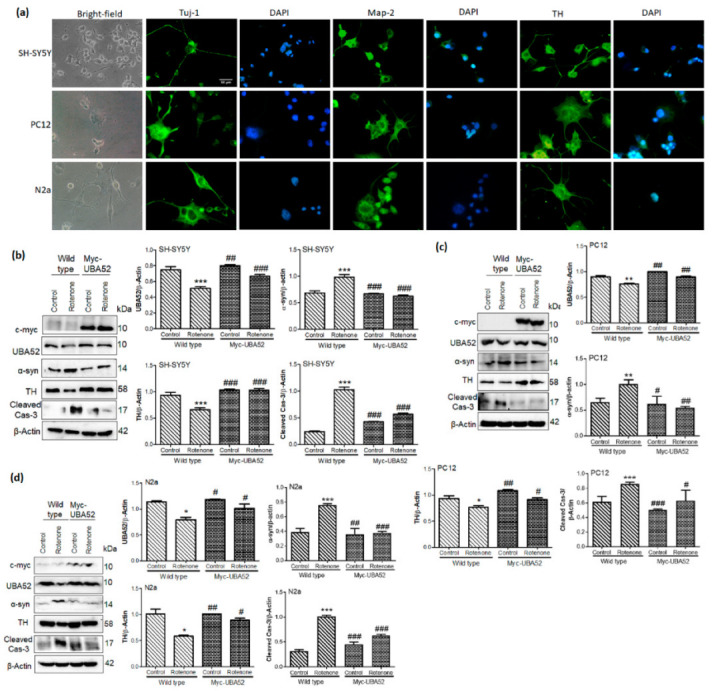
Effect of UBA52 overexpression on PD-related pathological markers in differentiated neuronal cell lines. (**a**) Representative image depicting the bright-field (20x) and immunofluorescence (40x) of differentiation markers Tuj-1 and Map-2 and dopaminergic marker tyrosine hydroxylase (TH) in SH-SY5Y, PC12 and N2a cells, scale bar—50μm. (**b**–**d**) Blots and graphical representations illustrating the PcDNA3.1 or Myc-UBA52 transfected SH-SY5Y, PC12 and N2a differentiated cells to show the PD-related protein markers expression with or without rotenone treatment; n_exp_ = 3. Quantifications are the mean and SEM of at least three independent experiments, and statistical analyses were performed using two-way ANOVA, followed by Tukey’s-multiple comparison test. * *p* < 0.05, ** *p* < 0.01, *** *p* < 0.001 control vs. Rotenone; # *p* < 0.05, ## *p* < 0.01, ### *p* < 0.001 Myc-UBA52 vs. Rotenone.

Upon obtaining significant results in bridling the effect of transient overexpression of Myc-UBA52 for PD-specific pathological markers (TH and α-synuclein) in an experimental acute model of SH-SY5Y, we next studied the effect of UBA52 in reversing the rotenone-induced PD conditions in differentiated neuronal cells employing SH-SY5Y, N2a and PC12 cells. Cells when cultured in a respective differentiating medium, showed high expression of differentiating markers (Tuj1 and Map2) and dopaminergic neuronal markers (TH) in SH-SY5Y, N2a and PC12 cells (Supplementary Figure S4, Figure 3a). To note, ATRA treatment did induce some cell death in all the neuronal cells, as reported previously in various differentiation-inducing protocol studies, in contrast to NGF treatment in PC12 cells. After 7 days of differentiation in respective cell lines, we investigated the effect of UBA52 on PD-specific marker TH, α-synuclein and neuronal apoptosis (cleaved caspase-3). Analysis of immunoblots in both wild-type and UBA52 overexpressed neuronal cells revealed the significant preventive effect of UBA52 in rotenone-induced pathological conditions as assessed by protein level of TH, α-synuclein and cleaved caspase-3 (Figure 3b–d). Although ATRA has not been reported as the differentiation-inducing chemical in N2a cells to promote dopaminergic characteristics, astonishingly, our results showed an apparently high expression of TH after 7 days of ATRA treatments (Figure 3d).

### 3.4. Effect of UBA52 on PD-Specific Pathological Markers in Experimental In Vitro Chronic Model

**Figure 4:**
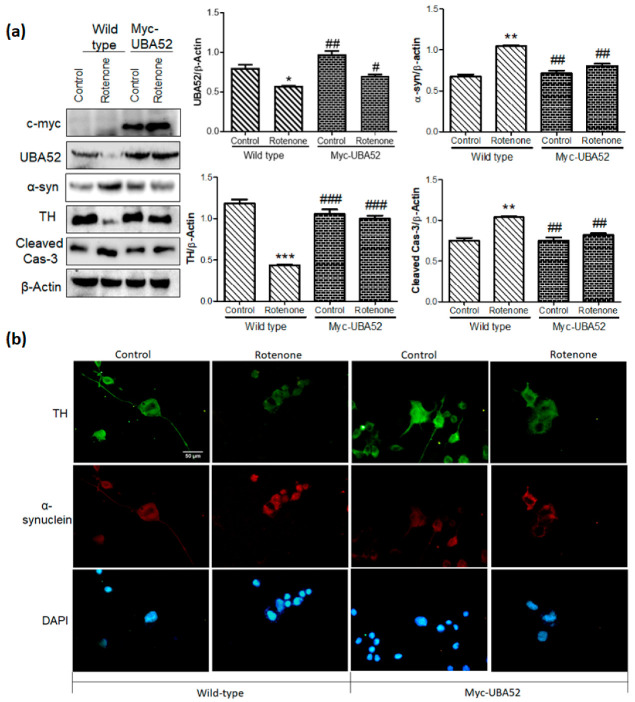
Effect of UBA52 overexpression on PD-related pathological markers in a chronic in-vitro model of SH-SY5Y cells. (**a**) Blots and graphical representations illustrating the PcDNA3.1 (control) or Myc-UBA52 transfected SH-SY5Y differentiated cells with or without chronic exposure of rotenone (50 nM) to show PD-related protein marker expression; n_exp_ = 3. (**b**) Representative image depicting immunofluorescence (40x) of TH and α-synuclein in PcDNA3.1 (control) or Myc-UBA52 transfected SH-SY5Y differentiated cells with or without chronic exposure of rotenone (50 nM) to show alteration in neuronal morphology, neurite length and expression of PD-related proteins, scale bar-50 μm; n_exp_ = 2. Quantifications are the mean and SEM of at least three independent experiments, and statistical analyses were performed using two-way ANOVA, followed by Tukey’s multiple comparison test. * *p* < 0.05, ** *p* < 0.01, *** *p* < 0.001 control vs. Rotenone; # *p* < 0.05, ## *p* < 0.01, ### *p* < 0.001 Myc-UBA52 vs. Rotenone.

The study was further expanded to assess the effect of UBA52 on a chronic experimental model of PD employing SH-SY5Y cells [29]. To aim this, we differentiated the SH-SY5Y cells for 7 days and then exposed the differentiated dopaminergic cells to rotenone (50 nM) for 14 days (12 days rotenone + 48 h of Myc-UBA52 transient expression) to develop chronic PD in vitro model. Rotenone treatment induced the neurite swellings from day 5 with a gradual decrease in neurite processes and cell viability till day 14 (data not shown). Immunoblotting data suggested significant alteration in the protein level of TH, α-synuclein and cleaved caspase-3 levels after rotenone treatment in chronic in vitro SH-SY5Y cells, which was discernibly reduced upon transient expression of Myc-UBA52 with or without rotenone exposure (Figure 4a). The cleaved caspase-3 protein level was also observed in control and Myc-UBA52 expressing cells, which accounted for both long-duration maintenance of SH-SY5Y cells and the initial 7 days of ATRA treatment; however, it was less in comparison to treated cells (Figure 4a). In parallel, we also confirmed our findings through immunofluorescence of TH and α-synuclein in a chronic in vitro model. Similar to immunoblotting, chronic rotenone treatment reduced the neurite processes in comparison to control cells, which were relatively less in cells transiently expressing Myc-UBA52 with or without rotenone exposure (Figure 4b). Due to severe loss of cell viability, as evident through observed cleaved caspase-3 level in control cells, the study was restricted to 14 days only. The observed findings in chronic pathological conditions are in concurrence with the findings of the acute model and suggest that UBA52 is a relevant pathophysiological regulator during PD.

### 3.5. Tuning the Interaction between HSP90 and CHIP by UBA52

**Figure 5:**
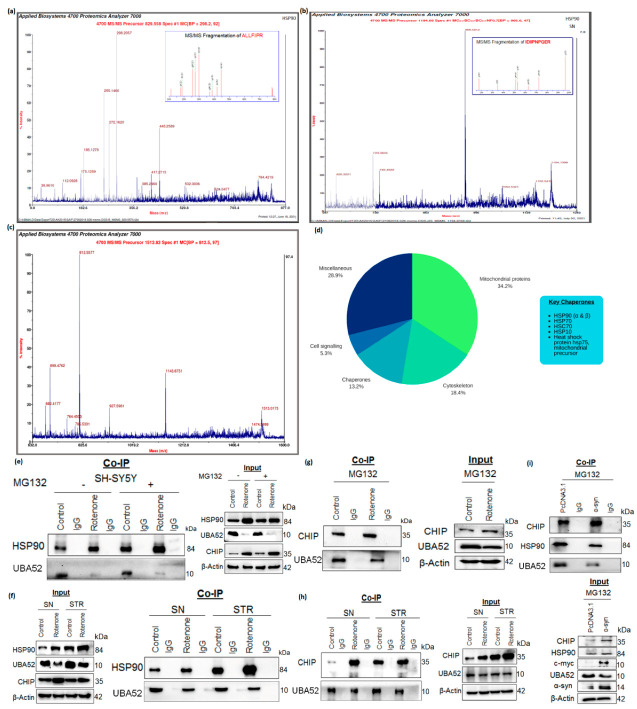
UBA52 interacts with HSP90 and CHIP. Co-IP (Protein A Sepharose beads attached to the anti-UBA52 antibody/anti-IgG antibody) was performed, and the cell or tissue lysates were incubated with the antibody-beads complex, following which the beads were boiled with 2X-reducing buffer, centrifuged and the supernatant was resolved on a 4–16% gradient SDS-PAGE. The gel was stained with Coomassie, followed by distaining and band excision to identify the interacting proteins through mass-spectrometric-based detection. MS/MS peaks representing the fragmentation spectrum (highest intensity) obtained from the tryptic peptides of amino acids of HSP90 α/β in (**a**) SH-SY5Y cells and (**b**,**c**) in rat brain regions; n_exp_ = 3. (**d**) The pie diagram illustrates the key chaperones obtained from the mass-spectrometric analysis. (**e**) Immunoblots representing the endogenous UBA52 and HSP90 with or without rotenone and MG132 treatment in SH-SY5Y cells; n_exp_ = 3 and (**f**) in control and rotenone-lesioned SN and STR rat brain region; n_exp_ = 4 after the Co-IP was performed using Protein A Sepharose beads and anti-UBA52 antibody/anti-IgG antibody complex incubated with cell and tissue lysates. (**g**) Next, Co-IP (Protein A Sepharose beads attached to the anti-UBA52 antibody/anti-IgG antibody) was utilized to detect the interaction of endogenous UBA52 and E3 ubiquitin ligase CHIP in SH-SY5Y cells with or without rotenone treatment with MG132, along with input bands n_exp_ = 3. (**h**) Immunoblots representing the interaction of endogenous UBA52 and CHIP in control and rotenone-treated rat brain regions, substantia nigra (SN) and striata (STR) n_exp_ = 3 after Co-IP with an anti-UBA52 antibody/anti-IgG antibody complexed with Protein A Sepharose beads. (**i**) Further, the SY-SY5Y cells were transiently overexpressed with Myc-α-synuclein (α-syn) to induce a pathological state. Next, the interaction of endogenous UBA52 with CHIP and UBA52 with HSP90 was assessed through immunoblotting after the Co-IP was performed using the complex of Protein A Sepharose beads and anti-UBA52 antibody/anti-IgG antibody incubated with PcDNA3.1 or Myc-tagged α-syn transfected SH-SY5Y cells after MG132 treatment; n_exp_ = 3.

In view of transiently overexpressed UBA52-mediated neuroprotection upon rotenone treatment, we searched for the novel interacting partners of UBA52 that might act as a substrate for ubiquitylation in the presence of putative E3 ligases. To accomplish this, we performed MALDI-TOF (matrix-assisted laser desorption ionization-time of flight)-based mass spectrometric (MS) analysis, with the aim of detecting the UBA52-interacting proteins in both cell and tissue lysates. Interestingly, the series of hits obtained and data analyzed by MASCOT software suggested the interaction of various proteins with UBA52. However, this study was focused on the interacting chaperones owing to their critical role in protein degradation mechanisms, and henceforth, the data were sorted accordingly. Findings suggested a strong interaction of UBA52 with HSP90 (Figure 5a–c and Figure S5), which was validated additionally by co-immunoprecipitation using anti-UBA52 antibody and IgG as control. Previously, it has been reported that HSP90 is ubiquitylated by E3 ubiquitin ligase CHIP (carboxy terminus of HSC70 interacting protein) and degraded by the proteasome [53]; therefore, we checked the association of UBA52 and CHIP. Co-immunoprecipitation of lysates using anti-UBA52 antibody showed the high interaction of CHIP after rotenone treatment, suggesting considerable participation of UBA52 in HSP90 ubiquitylation during disease induction (Figure 5d–f).

To further confirm the specificity of UBA52 in pathological conditions, the SH-SY5Y cells were transiently overexpressed with wild-type α-synuclein (Myc-α-SYN) to imitate the PD conditions, and the interaction of UBA52 with HSP90 and CHIP was evaluated. Overexpression of α-synuclein in neuronal cells significantly enhanced the interaction of UBA52 with HSP90 and CHIP (Figure 5g). Simultaneously, we also observed the decreased level of UBA52 (input band) after α-synuclein overexpression (Figure 5g), reaffirming our data of depleted UBA52 upon rotenone exposure.

**Figure 6:**
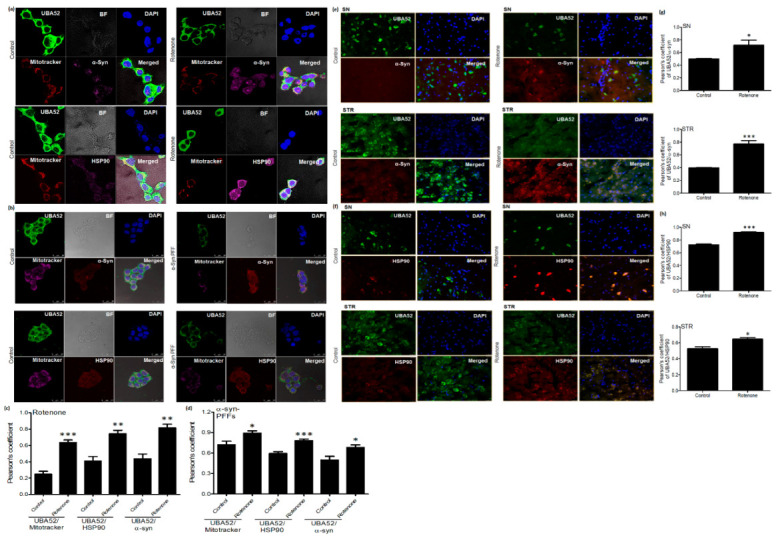
UBA52 co-localizes with α-syn and HSP90. (**a**,**c**) Confocal microscopy images and Pearson’s correlation coefficient analysis represent the co-localization of UBA52 and α-syn/HSP90 in control and rotenone-treated SH-SY5Y cells along with the mitotracker deep red (647 nm) and DAPI (405 nm) representing the mitochondrion and nucleus, respectively, Alexa fluor: Green-488, Pink-546; n_exp_ = 3. (**b**,**d**) Confocal microscopy images and Pearson’s correlation coefficient analysis represent the co-localization of UBA52 (Alexa fluor Green-488) and α-syn/HSP90 (Alexa fluor Red-546) in control and human recombinant α-synuclein preformed fibrils (PFFs)-induced SH-SY5Y cells along with the mitotracker deep red (647 nm) and DAPI (405 nm) representing the mitochondrion and nucleus, respectively, scale bar—25 μm; n_exp_ = 2. (**e**,**g**) Fluorescent images of rat brain sections representing the co-localization of UBA52 (Alexa fluor Green-488) and α-syn (Alexa fluor Red-546) in both SN and STR regions; n_exp_ = 3. (**f**,**h**) Fluorescent images of rat brain sections representing the co-localization of UBA52 (Alexa fluor Green-488) and HSP90 (Alexa fluor Red-546) in both SN and STR regions; n_exp_ = 3. Quantifications are the mean and SEM of at least three independent experiments, and statistical analyses were performed using Student’s *t*-test. (**c**). * *p* < 0.05, ** *p* < 0.01, *** *p* < 0.001 control vs. treated.

The last two years’ findings also highlighted that both α-synuclein and HSP90 are re-localized to the mitochondrion within the neuronal cell during PD progression [19]. Since UBA52 is ubiquitously expressed in the cytoplasm of the neuronal cell, we checked whether it co-localizes with HSP90 and α-synuclein along with its own translocation to other cellular organelles/membranes within the cell following disease induction. Confocal microscopy imaging in SH-SY5Y cells suggested that disease onset enables UBA52 localization in the mitochondrion (Figure 6a,b). Findings also revealed that UBA52 was highly co-localized with HSP90 and α-synuclein in both rotenone-treated and α-Syn PFF-induced SH-SY5Y cells in the cytosol as well as the mitochondrion in comparison to the control cells (Figure 6a,b). Last, we also validated our above findings of SH-SY5Y cells and checked the co-localization of UBA52 with HSP90 and α-synuclein in both SN and STR regions of the rat brain. Microscopic examination revealed a very strong co-localization of UBA52 with α-synuclein as well as with HSP90 in the rotenone-induced PD model of rats in comparison to the respective control (Figure 6c,d). Altogether, these approaches confirmed the protein-protein interaction of UBA52 with the molecular chaperone HSP90, E3 ligase CHIP and unequivocally linked PD marker α-synuclein.

### 3.6. Role of UBA52 in HSP90 Ubiquitylation during Parkinson’s Disease

**Figure 7:**
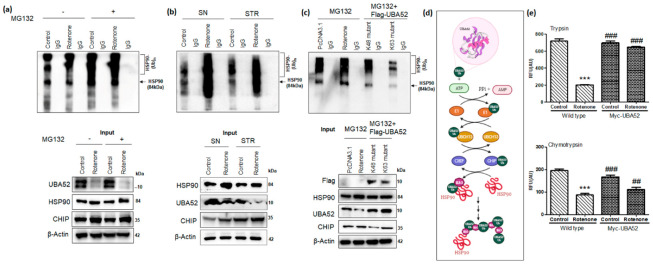
UBA52 is crucial for CHIP-mediated poly-ubiquitylation or multi-monoubiquitylation of HSP90 at lysine 63. Co-IP (Protein A Sepharose beads attached to the endogenous anti-UBA52 antibody/anti-IgG antibody) to analyze the interacting proteins, followed by in vitro ubiquitylation assay was performed, and the final reaction mixture was resolved on a 4–16% gradient SDS-PAGE and immunoblotted using an anti-HSP90 antibody. (**a**) Immunoblots probed after Co-IP and in vitro ubiquitylation assay of SH-SY5Y cell lysates-based final reaction mixture, against IgG negative control with or without MG132 and rotenone treatment; n_exp_ = 2 (**b**) Immunoblots represent the in vitro ubiquitylation assay after Co-IP of brain lysate of control and rotenone-lesioned SN and STR region of rat brain against endogenous anti-UBA52 antibody/anti-IgG antibody; n_exp_ = 2. The positive in vitro ubiquitylation reaction was observed in the presence of the E2 enzyme, UBCH13 in both (**a**) and (**b**) experimental studies. (**c**) Next, the SH-SY5Y cells were transiently overexpressed with mutant UBA52 (Flag-UBA52^K48A^ or Flag-UBA52^K63A^) to elucidate the specific lysine involved in ubiquitylation of HSP90 after MG132 treatment in control/rotenone treated cells, against IgG negative control; n_exp_ = 2. The data suggested the involvement of K63-linked ubiquitylation of HSP90. (**d**) Pictorial representation of CHIP-UBA52-UBCH13 mediated K63-linked ubiquitylation of HSP90. (**e**) Graphical representation for proteasome (trypsin and chymotrypsin) activity in wild-type SH-SY5Y cells and after transient overexpression of UBA52 (Myc-UBA52) with or without rotenone treatment; n_exp_ = 3. Quantifications are the mean and SEM of at least three independent experiments, and statistical analyses were performed using two-way ANOVA, followed by Tukey’s multiple comparison test. *** *p* < 0.001 control vs. Rotenone; ## *p* < 0.01, ### *p* < 0.001 Myc-UBA52 vs. Rotenone.

Since the protein-protein interaction of UBA52 with HSP90 and CHIP was observed in pathological conditions, we next investigated the role of UBA52 in HSP90 ubiquitylation in consideration of the observed increased level of HSP90 upon rotenone treatment and probable therapeutic implication of HSP90 in PD [54]. In vitro ubiquitylation assay in cells and in rat brain lysates suggested the increase in total ubiquitylation of HSP90 after rotenone administration along with an increase in the protein level of CHIP (Figure 7a,b). Furthermore, immunoblot probed with anti-HSP90 showed that in SN, the ubiquitylation was apparently more in comparison to the striatum region (Figure 7b), and the finding is in concordance with the location of the cell body in SN and nerve terminals in the STR. In vitro ubiquitylation assay was performed using various E2 enzymes, such as UBCH1, UBCH3, UBCH5c, UBCH7, UBCH10 and UBCH13. The E2 enzymes, UBCH5c and UBCH13 tag the protein at positions K48 and K63, respectively [55]. Of all the used E2 enzymes, UBCH13 showed its relevant involvement in ubiquitylation. Since ubiquitin labeling to target protein takes place through lysine residue, further investigation was performed to assess the specific lysine residue involved in the ubiquitylation of HSP90. To attain this aim, we transfected the SH-SY5Y cells transiently with vector encoding Flag-UBA52^K48R^ or Flag-UBA52^K63R^ mutants separately. Immunoprecipitation followed by ubiquitylation analysis showed that K63 mutation in UBA52 prevented the attachment of ubiquitin linkage on the HSP90 protein, which led to no change in total ubiquitin as well as total ubiquitylation of HSP90 in comparison to the results generated after mutation in K48 residue of UBA52 (Figure 7c). However, we did not spot similar results of ubiquitylation with α-synuclein as the target protein, confirming the findings from Tofaris et al. 2011 that CHIP and UBA52 (our findings) do not attach ubiquitin chain on α-synuclein in the presence of either UBCH5 (K48 linked) or UBCH13 (K63 linked) E2 ligases [56]. Taken together, our data strongly suggested that E3 ubiquitin-protein ligase CHIP ubiquitylates the target substrate HSP90 through E2 enzyme UBCH13, attaching K63 linked chains in the presence of ubiquitin gene UBA52 (Figure 7d).

Protein aggregates accumulate mainly due to a decrease in cellular degradation efficiency and decreased proteasome activity with aging or disease onset [57]. We, therefore, checked the proteasome activity in neuronal cells and in rat brain regions during the PD condition. Data indicated significant suppression of proteasome activity after rotenone treatment in both SH-SY5Y cells (Figure 7e), as well as in SN and STR regions of rat brains (Supplementary Figure S6). Subsequently, we transfected the neuronal cells with Myc-UBA52 and estimated its effect on the proteasomal (trypsin and chymotrypsin) activity. Findings suggested that wild-type UBA52 overexpressed cells resisted the decrease in proteasome activity upon neurotoxin exposure, and this observation was starker in trypsin as compared to chymotrypsin activity (Figure 7e). In another set of experiments, we checked the alteration in total ubiquitin levels through immunoblotting after UBA52 knockdown in control SH-SY5Y cells as well as in rotenone-treated cells. Rotenone treatment in neuronal cells increased the total ubiquitin level, whereas transient transfection of siRNA-UBA52 in control cells led to depletion in total ubiquitin levels. The upregulated ubiquitin level in rotenone-treated cells might be due to the activation of various downstream neurodegenerative mechanisms, which together increase the cargo load on the UPS to clear the aggregate-prone proteins, thereby raising the total ubiquitin level during the early phase of disease onset (Supplementary Figure S7). However, this observation was made in neuronal SH-SY5Y cells and is incomparable to human or rat physiological/pathological conditions. Therefore, it needs further investigation in more relevant genetically modified or AAVs-induced disease models. We propose that the observed increase in the level of ubiquitin during rotenone treatment may be transitory, and a time-dependent study would shed light in this direction. Altogether, UBA52 overexpression in neuronal cells prevents the lapse in proteasome activity during PD onset, therefore, maintaining the protein turnover and inhibiting protein aggregation.

### 3.7. Effect of UBA52 on Client Proteins of HSP90 and ER Stress-Related Pathophysiological State

**Figure 8:**
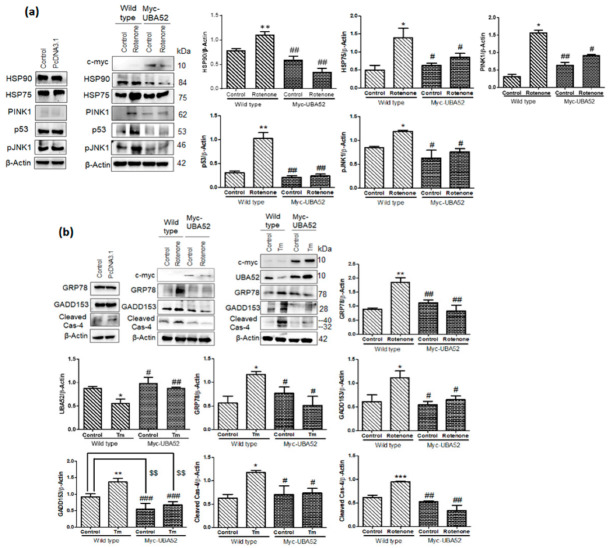
HSP90 client proteins and ERS (ER stress) marker protein expression in UBA52 overexpressed SH-SY5Y cells. (**a**) SH-SY5Y cells were transfected with PcDNA3.1 or Myc-UBA52 with or without rotenone treatment, and protein expression of client proteins of HSP90 was immunoblotted with indicated antibodies; n_exp_= 3. (**b**) Blots represent the protein expression of ER stress markers in PcDNA3.1 or Myc-UBA52 transfected SH-SY5Y cells with or without rotenone or tunicamycin (Tm) treatment. Graphs indicate the statistical analysis of various proteins normalized against β-Actin; n_exp_ = 3. Quantifications are the mean and SEM of at least three independent experiments, and statistical analyses were performed using two-way ANOVA, followed by Tukey’s multiple comparison test. * *p* < 0.05, ** *p* < 0.01, *** *p* < 0.001 control vs. Rotenone; # *p* < 0.05, ## *p* < 0.01, ### *p* < 0.001 Myc-UBA52 vs. Rotenone; $$ *p* < 0.01 control vs. Myc-UBA52.

Given the powerful ability of Myc-UBA52 in protecting SH-SY5Y cells against the altered expression of PD-specific pathological markers, we further checked the effect of UBA52 on the protein level of HSP90 in SH-SY5Y cells. Myc-UBA52 significantly reduced the augmented protein level of HSP90, and this reduction was more prominent in the rotenone-induced Myc-UBA52 group and maybe in order to prohibit neurodegeneration (Figure 8a). This finding is in agreement with a previous report by [54], suggesting that HSP90 inhibition may be a potential target in treating a diverse array of neurodegenerative diseases.

HSP90 associates with and stabilizes more than 200 client proteins, such as p53 (cell-cycle), PINK1, AKT1, JNK1 and others, whose expression is altered during PD pathogenesis [58,59,60]. Furthermore, proteins such as HSP75/TRAP1 (an isoform of HSP90 in mitochondria) and PINK1 participate in mitochondrial function and bioenergetics, which are severely compromised during PD [61]. In this context, we have observed the increased level of PINK1, p53, pJNK1 and HSP75/TRAP1 in neuronal cells upon neurotoxin exposure, which was inhibited in Myc-UBA52 transfected cells even after PD induction, suggesting the involution of UBA52 in HSP90 and its client proteins interaction during PD onset (Figure 8a). In view of the significant investigation of ER stress signaling in PD pathogenesis [4,7,9,11,12], further study was expanded to assess the effect of UBA52 on ER stress-related signaling factors such as GRP78, GADD153, cleaved caspase-4 (human analog of caspase-12) in control and Myc-UBA52 transfected cells upon PD induction. Transient expression of Myc-UBA52 in neuronal cells inhibited the PD-related upregulated level of ER stress markers (Figure 8b). Moreover, as a positive control, we performed a similar set of experiments using tunicamycin (ER stress inducer) to study the possible role of UBA52 in ER stress-related signaling. As shown in Figure 8b, no evident difference was observed in Myc-UBA52 transfected neuronal cells upon tunicamycin treatment compared to only tunicamycin-treated neuronal cells. Findings indicated that UBA52 overexpression protects the neuronal cells against UPS initiation and chaperone-related PQC after rotenone treatment and may have therapeutic implications.

## 4. Discussion

Considering protein misfolding as a prime pathology in PD pathogenesis, the available information regarding UPS and its regulatory components (ubiquitin and ubiquitin ligases) is limited. Ubiquitin ligases are essential in the mandatory tagging of ubiquitin to target proteins for their processing to degradation via the proteasome. To date, a large number of ubiquitin ligases have been reported but their known participation in PD pathology is scarce. In PD pathology, the important role of Parkin, CHIP, E6AP, SIAH, ITCH, and TRIM2/9 has been reported [2,62,63] and in confined test systems. Although the function of E3 ligase Parkin is extensively studied in association with PINK1 to regulate energy biogenesis [64,65,66], the role of the core component of UPS in ubiquitin has not been yet reported. In this study, for the first time, we report the significant role of UBA52 in PD pathology during the early phase of the disease. Additionally, we divulge the participation of UBA52 in CHIP-mediated ubiquitylation of a molecular chaperone, HSP90, and finally, decipher the role of UBA52 in orchestrating the associated neurodegenerative signaling. Separately, we showed that transient overexpression of UBA52 in neuronal cells provides protection against the onset of PD pathology, highlighting its critical partaking in the initiation of the death mechanism, probably due to protein misfolding and insufficient chaperoning, eventually protecting the dopaminergic neurons.

Our findings in human Myc-α-synuclein transfected neurons, α-synuclein-PFFs treated SHSY5Y cells, rotenone-induced sporadic cellular and rat models of PD and SNCA transgenic mice highlight that UBA52 is downregulated during the early phase of sporadic PD, which is critical and might account for the reduction in proteasome activity during the pathological condition. UBA52, on one hand, interacts with an important PD-related pathological protein, α-synuclein, and inhibits its upregulation to regulate its level to impede fibril formation. On the other hand, overexpression of UBA52 in both proliferative dopaminergic SH-SY5Y cells and differentiated SH-SY5Y, N2a and PC12 neuronal cells avert the downregulation of TH and caspase-3 activation. These findings raise the possibility that UBA52 has a perspicacious role in the early progression of PD-related pathogenesis. Our previous study on the role of ER stress [4,11] and on reduced proteasome activity in PD pathology [12] advocated the probable role of the molecular chaperone, as both ER functioning and PQC are closely regulated by chaperones in neuronal cells [67]. Among several chaperones, HSP90 has emerged as a novel therapeutic target for PD treatment [54]. In concurrence with this, our quantitative mass spectrometric and co-immunoprecipitation data demonstrated that UBA52 interacts with HSP90 and CHIP, forming a complex, wherein we suggested that E3 ligase CHIP adds a K63-mediated ubiquitin chain to HSP90 in the presence of UBA52. K63-mediated ubiquitylation is reported to be associated with various post-translational modifications such as endocytosis, signal transduction and autophagy [68]. It has been previously reported that K63-mediated ubiquitylation also reduces the cargo stress from proteasome, facilitates the clearance of inclusion bodies, and supports mitochondrial homeostasis [69], supporting our findings in the context of UBA52 overexpression-mediated neuroprotection. Despite the downregulation of UBA52 levels during the acute phase of disease onset, we propose that CHIP-UBA52 allied K63-linked HSP90 ubiquitylation spikes during sporadic PD conditions due to the increase in protein misfolding and HSP90 protein levels and to maintain the homeostatic protein turnover, highlighting the indispensable role of UBA52 in maintaining HSP90 levels. However, the progression of disease and initiation of other disease-related neurodegenerative signaling mechanisms tardily leads to cell death and needs to be investigated in detail. In agreement with our previous finding, a recent study has shown that the proteasome hydrolytic activity reduces during PD pathology, acting either as a cause or consequence to further exacerbate the disease pathology [13]. Protection against reduced proteasomal activity after rotenone exposure in the presence of overexpressed UBA52 also reflects its burgeoning part in sufficient degradation of target proteins such as HSP90 and impedes neurodegeneration during early PD. Our most surprising study is that control neuronal cells in which UBA52 was transiently silenced had a reduced total ubiquitin amount. However, neuronal cells expressing disease markers after exposure to the neurotoxin compensated for the loss of UBA52 with a high level of total ubiquitin content. In view of this unexpected finding, we assume that the onset of disease increases the cargo load on the proteasome, which increases the total ubiquitin level to temporarily escalate the protein turnover in order to restore proteostasis, compensating for the loss of UBA52. Although our study was conducted in the acute phase of PD pathology conditions, we observed that the proteasome activity was downregulated, analogous to upregulated ubiquitin levels during disease onset. Therefore, a detailed time-dependent study may shed light on this context. Since UBA52 depletion is observed at the early phase of disease pathogenesis, the diagnostic aspect of UBA52 could also be explored, though it may need excellent technical tools and specific dyes. However, it is an apparently achievable target on the account of an investigation regarding its peripheral level in the blood, urine and cerebrospinal fluid. Recently, Xu et al. 2021 [70] have also suggested the clinical significance of UBA52 level in urine samples for the diagnosis of diabetes mellitus and diabetic nephropathy.

Our findings related to augmented HSP90 protein abundance during PD are coherent with previous findings underlining that the HSP90 inhibitor, Geldanamycin (GA), potentially reduces the α-synuclein oligomer and aggregate formation as well as induces α-synuclein clearance [71]. Similarly, the other HSP90 inhibitor 17-AAG (17-allylamino-17-demethoxygeldanamycin, or Tanespimycin) also reduces the α-synuclein toxicity and facilitates its clearance in various experimental models of PD [71]. Consistent with these research findings and our data that UBA52 surplus in neuronal cells resists the upregulated level of HSP90, we proffer UBA52 as a promising candidate for addressing PD and Parkinsonism-related disorders. Furthermore, our findings affirm the previous findings on HSP90 inhibition, suggesting that HSP90 co-localizes with α-synuclein, and UBA52 surplus forestalls the protein aggregation by interacting with both the aforementioned proteins and regulating their levels.

To broaden the research arena, we also evaluated the level of a few bona fide client proteins of HSP90 that participate in PD-associated various neurodegenerative signals, such as pJNK1, p53, and PINK1 (PARK6). Myc-UBA52 transfected neuronal cells inhibited the change in the protein level of studied client proteins, suggesting that UBA52 has a salient role in pathways related to cell cycle arrest, apoptosis, autophagy and mitochondrial homeostasis. Mitochondrial chaperone, HSP75/TRAP1, regulates the formation of reactive oxygen species (ROS) and shows an explicit connection with PINK1, wherein PINK1 phosphorylates HSP75 to protect the neuronal cells against oxidative stress [72]. Findings showed insignificant alteration in the protein level of HSP75 after exposure to rotenone in Myc-UBA52 transfected cells. Altogether, our data point out that UBA52 implicitly or explicitly regulates mitochondrial functioning and has a multi-facet role in maintaining cellular machinery to prevent the onset of disease. Our confocal microscopy-based observations also showed localization of UBA52 in the mitochondrion during disease onset, reaffirming the implications of UBA52 in mitochondrial homeostasis. Since protein homeostasis is essential for cellular structure and function, protein misfolding or unfolding (as observed in PD pathology and other neurodegenerative diseases) intends to activate the cellular PQC involving various molecular chaperones and UPS [73]. It is evident from our generated data in the cellular and rodent PD model that UBA52 surfeit reduces the proteotoxic stress and regulates the ER functionality, as evidenced by the insignificant change in the protein level of ER stress markers upon exposure to the neurotoxin to prevent neuronal death.

In summary, our findings propose a conspicuous role of UBA52 in maintaining the E3 ubiquitin ligase CHIP-mediated ubiquitylation of chaperone HSP90, along with the subsequent effect on its important client proteins, thereby regulating multiple pathways simultaneously during PD pathogenesis (graphical abstarct).

Such diverse roles of UBA52 should be explored further in the context of its diagnostic, pharmacological and translational aspects. Though the study was focused on human Myc-UBA52 transfected neurons and rat models of PD and the SNCA transgenic mice, the limitation of the current study is the non-availability of a UBA52-specific transgenic animal model to assess its effect on PD pathology, especially the intracellular signaling pathways and mitophagy/autophagy, which are very challenging and subject to future investigation. Nevertheless, our study claims that UBA52 displays significant efficacy in orchestrating various signaling mechanisms related to dopaminergic neuronal death, in view of its direct interference in regulating the α-synuclein level and ubiquitylation of HSP90 in complex with CHIP to maintain the efficient chaperoning. In addition, other neurodegenerative diseases especially linked to synucleinopathies and protein aggregation in which the therapeutic implications of HSP90 inhibitors have been suggested, may perhaps consider UBA52 as a promising candidate for targeting disease therapeutics and mitigating neuronal death.

## Data Availability

All the generated data are compiled and given in MS or Supplementary files. The raw data will be provided upon reasonable request.

## Supplementary Materials

The following supporting information can be downloaded at: https://www.mdpi.com/article/10.3390/cells11233770/s1.

## Abbreviations

PD	Parkinson’s disease
SN	Substantia nigra
STR	Striatum
TH	Tyrosine hydroxylase
UPS	Ubiquitin–proteasome table system
ERS	Endoplasmic reticulum stress
UBA52	Ubiquitin-60S ribosomal protein L40
UBB	Polyubiquitin-B
UBC	Polyubiquitin-C
RPS27a	Ubiquitin-40S ribosomal protein S27a
RT-qPCR	Real-time quantitative PCR
MS	Mass spectrometry
MALDI-TOF	Matrix-assisted laser desorption ionization-time of flight
ACN	Acetonitrile
ABC	Ammonium Bicarbonate
TFA	Trifluro acetic acid
IgG:	Immunoglobulin G
PQC	Protein quality control
PFFs	Preformed fibrils
PSOPIA	Prediction server of protein–protein interaction
HSP90	Heat shock protein 90
CHIP	C-terminus of HSC-70
K^48^R	Lysine residue mutated to arginine at position-48 of UBA52
K^63^R	Lysine residue mutated to arginine at position-63 of UBA52
pJNK1	phospho- c-Jun N-terminal kinase 1
AKT1	RAC-alpha serine/threonine-protein kinase 1
PINK1	PTEN-induced kinase 1
HSP75	Heat shock protein 75
