# Supplementary material for: UBA52 Is Crucial in HSP90 Ubiquitylation and Neurodegenerative Signaling during Early Phase of Parkinson’s Disease
Source: Cells. 2022 Nov 25;11(23):3770. doi: 10.3390/cells11233770 (PMC9738938; doi:10.3390/cells11233770)
Supplement: Supplementary file 1 [file cells-11-03770-s001.zip › cells-1993131-SM.pdf]

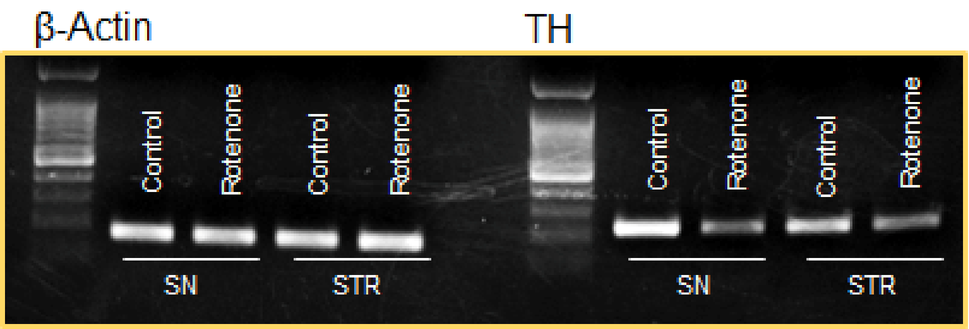

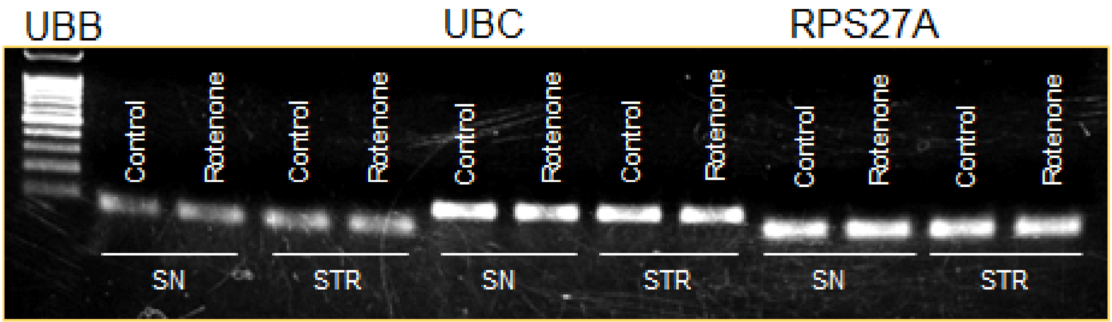

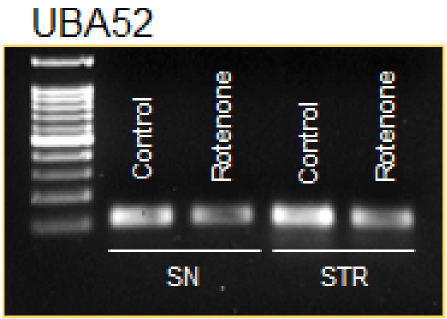

Supplementary Figure S1: Images showing the mRNA expression of β-Actin, TH (tyrosine hydroxylase), UBB (polyubiquitin-B), UBC (polyubiquitin-C), RPS27A (ribosomal protein S27a) and UBA52. The substantia nigra (SN) and striatum (STR) regions were isolated from control and diseased (rotenone treated) rat brain and tissue was processed for RT-PCR and agarose gel electrophoresis. $n_{rats}=4$; $n_{exp.}=3$.

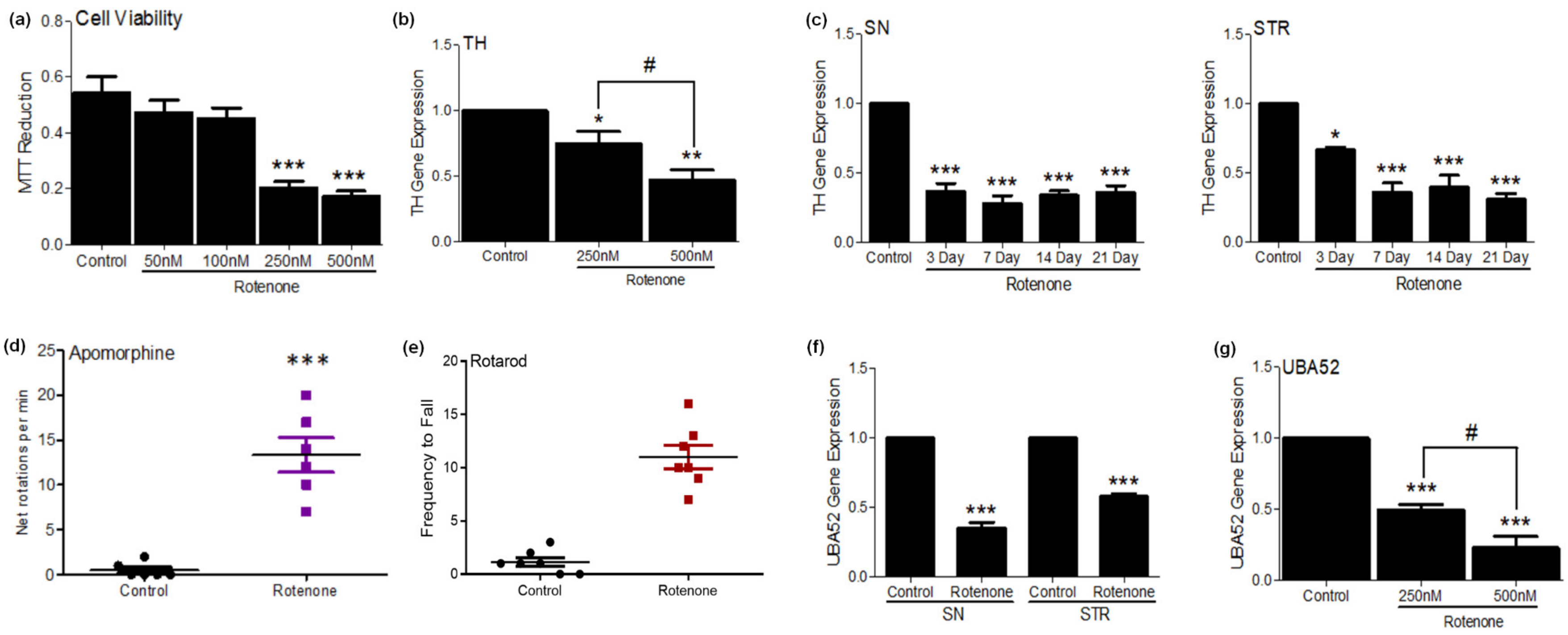

Supplementary Figure S2: Parkinson's disease markers and UBA52 expression in SHSY5Y and rat brain (a) Graph representing MTT reduction assay in SH-SY5Y cells to confirm optimum treatment dose of rotenone. **(b)** qPCR of TH in SH-SY5Y cells and **(c)** SN (substantia nigra) & STR (striata) region of rat brain, normalized against β-Actin to determine time-point of study in the *in vivo* model after rotenone treatment; n=3. **(d & e)** Behavioral study of control and rotenone-lesioned rat. Contralateral rotations induced by apomorphine **(d)** and lapse in muscle-coordination measured through **(e)** rotarod test; n=3. **(f)** qPCR of UBA52 in control and neurotoxin-treated rat brain regions, normalized against β-Actin; n=4. **(g)** qPCR of UBA52, normalised against β-actin in SH-SY5Y cells after rotenone treatment; n=3. Colored bullets (black) and squares (purple and red) represent the total number of rats utilised during the study. Quantification are mean and SEM of at least three independent experiments and statistical analysis were performed using Student's t-test and one-way ANOVA. * $p<0.05$,** $p<0.01$, *** $p<0.001$ control vs. Rotenone; # $p<0.05$ R250nM vs. R500nM.

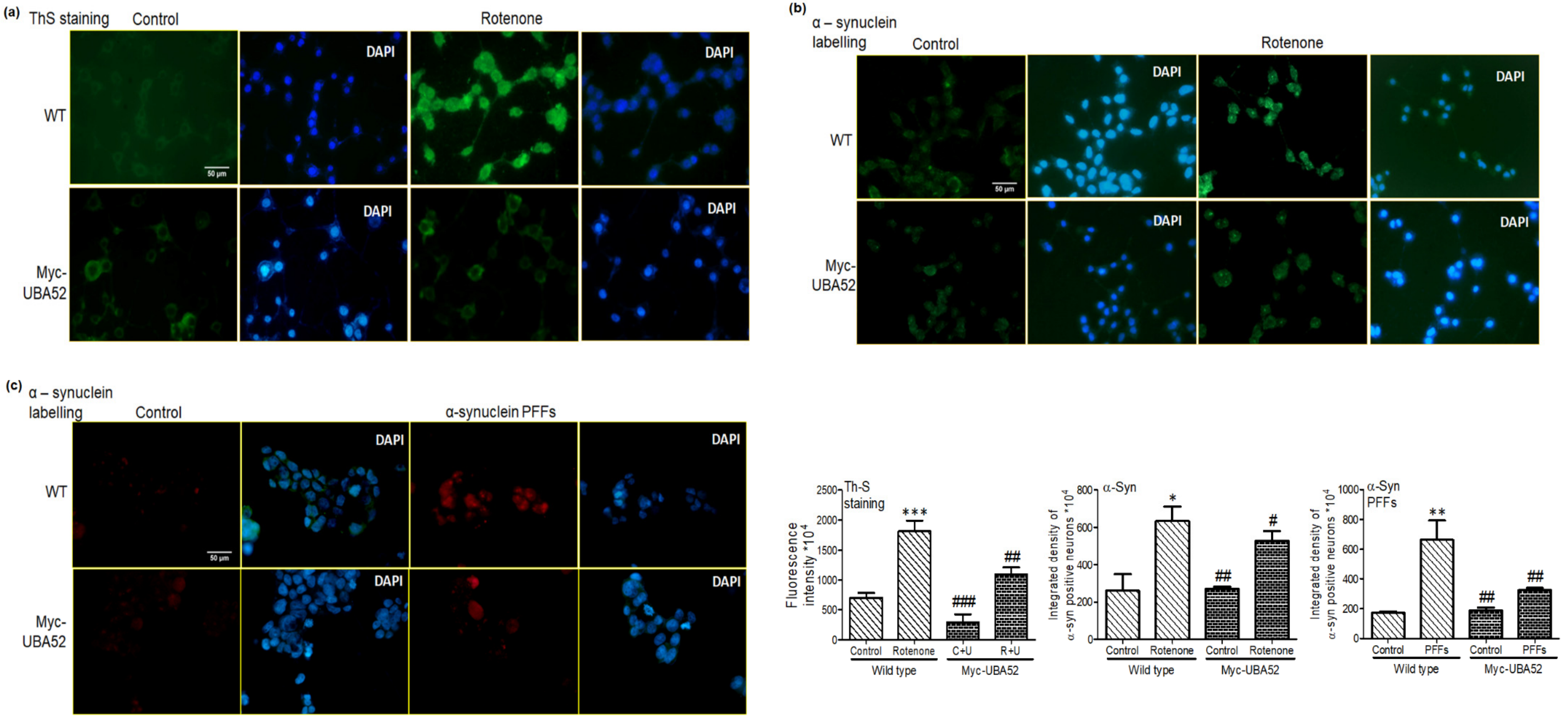

Supplementary Figure S3: UBA52 interacts with α-Syn and its over-expression in SH-SY5Y cells deter the alteration in PD markers protein abundance, preventing the disease onset. . **(a)** Thioflavin-S (Th-S) staining and the fluorescence intensity of Th-S positive neurons to detect protein aggregates in wild-type or Myc-UBA52 transfected SH-SY5Y cells with or without rotenone treatment, Scale bar-50μm; n_{exp}=3. **(b)** Florescent images and the integrated density depicting cytological staining for α-Syn positive neurons later tagged with Alexa fluor-488 (green) in wild-type or Myc-UBA52 transfected SH-SY5Y cells with or without rotenone treatment, Scale bar-50μm; n_{exp}=3. **(c)** Florescent images and the integrated density depicting cytological staining for α-Syn positive neurons later tagged with Alexa fluor-532 (red) in wild-type or Myc-UBA52 transfected SH-SY5Y cells with or without human recombinant α-Syn PFF treatment, Scale bar-50μm; n_{exp}=3. Quantification is the mean and sem of at least three independent experiments and statistical analysis was performed using two-way ANOVA, followed by Tukey's multiple comparison test. * p<0.05 , ** p<0.01, *** p<0.001 control vs. Rotenone/αSyn PFFs/Transgenic (Tg); # p<0.05 ,## p<0.01, ## p<0.001 Myc-UBA52 vs. Rotenone.

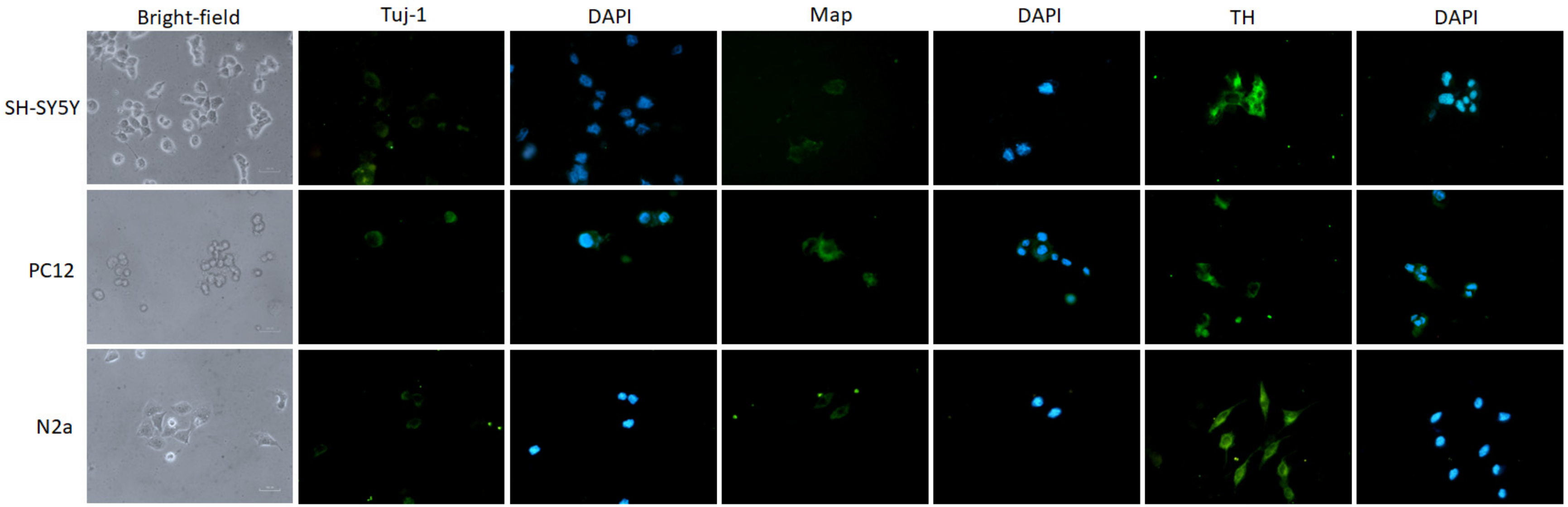

Supplementary Figure S4: Representative image depicting bright-field(20x) and immunofluorescence(40x) of differentiation markers Tuj-1, Map-2 and dopaminergic marker tyrosine hydroxylase (TH) in SHSY5Y, PC12 and N2a undifferentiated cells.

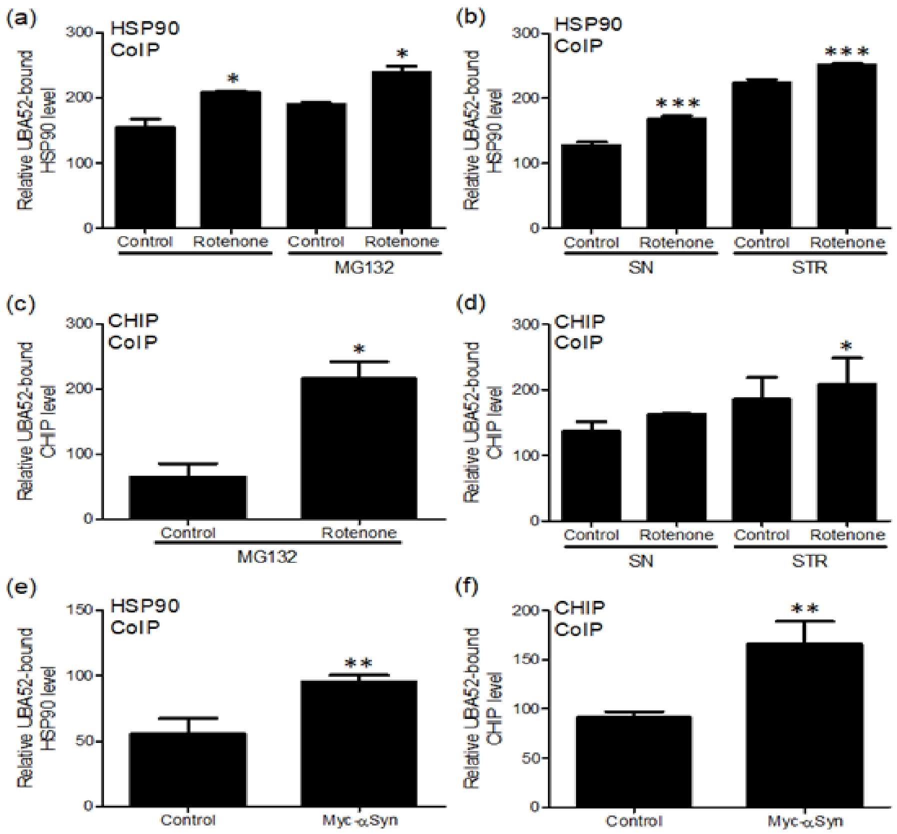

Supplementary Figure S5: Graphical representations depict the analyzed Co-IP immunoblots of the relative UBA52-bound (a) HSP90 level in SH-SY5Y cells with or without MG132 and rotenone treatment, (b) HSP90 level in the SN (substantia nigra) and STR (striata) region of the rat brain with or without rotenone treatment, (c) CHIP level in SH-SY5Y cells after MG132 treatment, (d) CHIP level in the SN and STR region of the rat brain with or without rotenone treatment, (e) HSP90 level after transient overexpression of Myc-αSyn, (f) CHIP level after transient overexpression of Myc-αSyn in comparison to the control. n_{rats}=4-5 per group per replicate; $n_{exp.}$=3-4. Quantification are mean and SEM of at least three independent experiments and statistical analysis were performed using unpaired t-test (for *in vivo* data) or two-way ANOVA, followed by Tukey's multiple comparison test (for *in vitro* data). * $p<0.05$, ** $p<0.01$ control vs Rotenone.

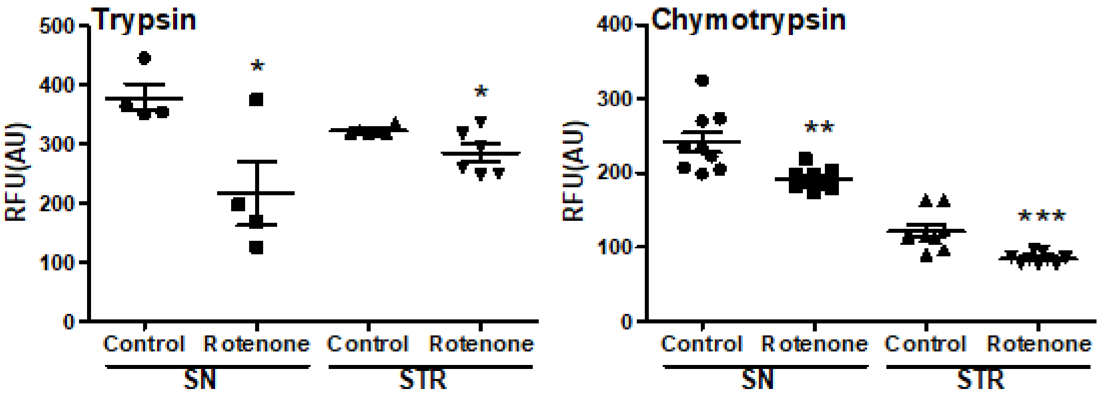

Supplementary Figure S6: Proteasome activity in SN and STR region of control and diseased (rotenone administered) rat brain. $n_{exp.}$=3, n_{rat}=4. The bullets, squares and triangles represent the number of total rats used in the study. Quantification is mean ± SEM of independent experiments and statistical analysis was performed using Student's t-test. * p<0.05, ** p<0.01, *** p<0.001 control vs. Rotenone.

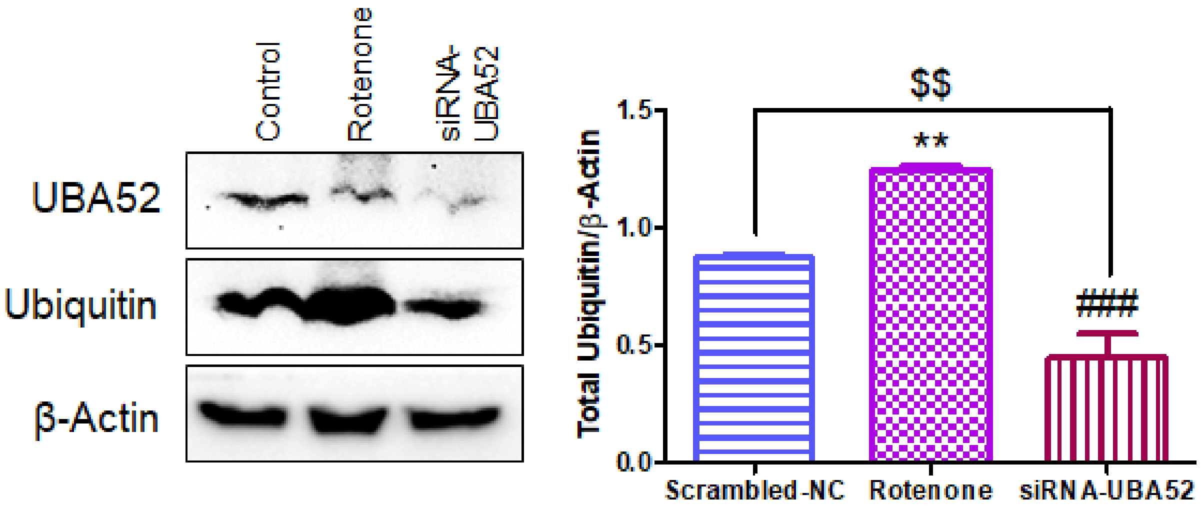

Supplementary Figure S7: Immunoblots and graphical representation of total ubiquitin levels after transfection of SH-SY5Y cells after rotenone treatment and transfection with siRNA-UBA52. $n_{exp.}=2$. Quantification are mean ± SEM of at least two independent experiments and statistical analysis were performed using two-way ANOVA, followed by Tukey's-multiple comparison test. ** $p<0.01$ Scrambled normal control (NC) vs. Rotenone; ### $p<0.001$ Rotenone vs. siRNA-UBA52; $$ $p<0.01$ Scrambled NC vs. siRNA-UBA52.

a.

PSOPIA Prediction Results

JOBID: D20210709_T150906_IP14-139-230-7_P1
DATE: Fri Jul 9 15:09:15 JST 2021

SEQUENCE1: sp|P62987|RL40_HUMAN Ubiquitin-60S ribosomal protein L40 OS=Homo sapiens OX=9606 GN=UBA52 PE=1 SV=2 Pfam
SEQUENCE2: sp|P37840|SYUA_HUMAN Alpha-synuclein OS=Homo sapiens OX=9606 GN=SNCA PE=1 SV=1 Pfam

S_{seq}	S_{dom}	S_{net}	S_{all}
0.8815	0.5146	0.8351	0.9123

PSOPIA
PREDICTION SERVER OF PROTEIN-PROTEIN INTERACTIONS

© PSOPIA Copyright

PSOPIA Prediction Results

JOBID: D20210709_T154503_IP14-139-230-7_P1
DATE: Fri Jul 9 15:45:14 JST 2021

SEQUENCE1: sp|P62987|RL40_HUMAN Ubiquitin-60S ribosomal protein L40 OS=Homo sapiens OX=9606 GN=UBA52 PE=1 SV=2 Pfam
SEQUENCE2: sp|P07101|TY3H_HUMAN Tyrosine 3-monooxygenase OS=Homo sapiens OX=9606 GN=TH PE=1 SV=5 Pfam

S_{seq}	S_{dom}	S_{net}	S_{all}
0.3613	0.0000	0.0000	0.3613

PSOPIA
PREDICTION SERVER OF PROTEIN-PROTEIN INTERACTIONS

© PSOPIA Copyright

b.

\# PSOPIA Prediction Results

JOBID: D20170804_T134657_IP14-139-230-12_P1
DATE: Fri Aug 4 13:47:07 JST 2017

SEQUENCE1: sp|P62986|RL40_RAT Ubiquitin-60S ribosomal protein L40 OS=Rattus norvegicus GN=Uba52 PE=1 SV=2 Pfam
SEQUENCE2: sp|P37377|SYUA_RAT Alpha-synuclein OS=Rattus norvegicus GN=Snca PE=1 SV=1 Pfam

S_{seq}	S_{dom}	S_{net}	S_{all}
0.7961	0.5146	0.8351	0.7731

PSOPIA
PREDICTION SERVER OF PROTEIN-PROTEIN INTERACTIONS

© PSOPIA Copyright

\# PSOPIA Prediction Results

JOBID: D20170804_T135549_IP14-139-230-12_P1
DATE: Fri Aug 4 13:55:59 JST 2017

SEQUENCE1: sp|P62986|RL40_RAT Ubiquitin-60S ribosomal protein L40 OS=Rattus norvegicus GN=Uba52 PE=1 SV=2 Pfam
SEQUENCE2: sp|P04177|TY3H_RAT Tyrosine 3-monooxygenase OS=Rattus norvegicus GN=Th PE=1 SV=3 Pfam

S_{seq}	S_{dom}	S_{net}	S_{all}
0.3537	0.0000	0.0000	0.3537

PSOPIA
PREDICTION SERVER OF PROTEIN-PROTEIN INTERACTIONS

© PSOPIA Copyright

Supplementary File S1: In-silico analysis indicating the interaction of UBA52 with TH and α-synuclein as assessed through a freely accessible server, PSOPIA (Prediction Server of Protein-Protein Interaction), based on both human **(a)** and rat **(b)** protein sequences available on UniProtKB database.

Supplementary Information:

Chemicals:
Bovine serum albumin (BSA), disodium hydrogen phosphate (Na_2HPO_4), dimethyl sulfoxide (DMSO), ethidium bromide, glucose,4-(2-hydroxyethyl)-1-piperazine ethane sulfonic acid (HEPES), 3-(4,5-dimethylthiazol-2-yl)-2,5- diphenyltetrazolium bromide dye (MTT), NP-40, phenylmethylsulphonyl fluoride (PMSF), magnesium chloride ($MgCl_2$), dithiothreitol (DTT), RNase, sodium bicarbonate and tris-buffer were procured from SRL, India. Dulbecco's modified Eagle's medium (DMEM), fetal bovine serum (FBS), Trizol, Ham's F12 medium, penicillin-streptomycin, nuclease-free water, lipofectamine 3000 and mitotracker-red and -deep red were purchased from Invitrogen (San Diego, CA, USA). Luria Bertani broth and agar powder were purchased from Himedia. *In vitro* ubiquitylation kit was purchased from Enzo-Life sciences. Recombinant human α-synuclein protein PFF (ab218819) and recombinant mouse α-synuclein protein PFF (ab246002) were purchased from Abcam. Other chemicals such as Protein-A Sepharose beads, anti-fade medium DAPI, copper sulfate ($CuSO_4$), calcium chloride ($CaCl_2$), Folin–Ciocalteu reagent, potassium chloride (KCl), sodium carbonate ($NaHCO_3$), sodium chloride (NaCl), sodium dihydrogen phosphate (NaH_2PO_4), sodium hydroxide (NaOH), protease and phosphatase inhibitor cocktail, rotenone, tunicamycin, apomorphine, paraformaldehyde (PFA), ethylenediaminetetraacetic acid (EDTA), acetonitrile (ACN), ammonium bicarbonate (ABC), trifluoro acetic acid (TFA) and trypsin MS grade were obtained from Sigma, USA.

Table S1: Antibodies used for Immunoblot (WB) and immunofluorescence (IF)

Antibodies	Company	Catalogue Number	Species	Dilution WB	Dilution IF
β-Actin	Sigma-Aldrich	A3854	Mouse	1:10000	
α-Synuclein	Santa Cruz Biotechnology	sc-53955	Mouse	1:500	1:100
TH	Sigma-Aldrich	T8700	Rabbit	1:1000	
TH	Sigma-Aldrich	T2928	Mouse	1:1000	1:250
UBA52	Abcam	ab109227	Rabbit	1:1000	1:250
HSP90	Cell Signalling	4874S	Rabbit	1:1000	
HSP90	Santa Cruz Biotechnology	sc-515081	Mouse	1:500	1:100

CHIP	Santa Cruz Biotechnology	sc-133066	Mouse	1:500	
pJNK	Santa Cruz Biotechnology	sc-6254	Mouse	1:500	
p53	Santa Cruz Biotechnology	sc-98	Mouse	1:500	
Cleaved Caspase-3	Invitrogen	700182	Rabbit	1:1000	
Cleaved Caspase-4	Santa Cruz Biotechnology	sc-56056	Mouse	1:500	
Ubiquitin	Cell Signalling	3936S	Mouse	1:1000	
Myc-Tag	Cell Signalling	2276S	Mouse	1:1000	
Flag-M2	Sigma	F1804	Mouse	1:1000	
HSP75	Santa Cruz Biotechnology	sc-13577	Rabbit	1:1000	
PINK1	Santa Cruz Biotechnology	sc-517353	Mouse	1:500	
GRP78	Abcam	ab21685	Rabbit	1:5000	
GADD153	Santa Cruz Biotechnology	sc-7351	Mouse	1:500	
Anti-Mouse Secondary	Sigma-Aldrich	A9044		1:5000	
Anti-Rabbit Secondary	Sigma-Aldrich	A0545		1:3000	
Alexa-fluor 488 Green	Invitrogen	A11034	Rabbit		1:300
Alexa-fluor 488 Green	Invitrogen	A11059	Mouse		1:300
Alexa-fluor 546 Red	Invitrogen	A11003	Mouse		1:300
Alexa-fluor 647 Deep Red	Invitrogen	A21235	Mouse		1:300

Table S2: List of primers sequences used for RT-PCR

Gene Name	cDNA Primer Sequences
β-actin	5' GTCGTACCACTGGCATTGTG 3'<br>3' CTCTCAGCTGTGGTGGTGAA 5'
TH (Human)	5' TGTGGCCTTTGAGGAGAAGGA 3'<br>3' TCAAACACCTTCACAGCTCGG 5'
TH (Rat)	5' CCA CGG TGT ACT GGT TCA CT 3'<br>3' GGC ATA GTT CCT GAG CTT GT 3'
UBA52 (Human)	5' AGATGATGCCAAAGGACGCA 3'<br>3' TCAATGGTGTCACTGGGCTC 5'
UBA52 (Rat)	5' GCAGACGCCAACATGCAGA 3'<br>3' GGGGATGCCTTCCTTGTCTT 5'
RPS27A (Rat)	5' GGTCTAATCCGTCTCTTTTC 3'<br>3' CTGGATCTTGGCCTTTACAT 5'
UBB (Rat)	5' TAGCCATTTGCCCCAATTTA 3'<br>3' TGCTTACCATGCAACAAAAC 5'
UBC (Rat)	5' ACCTTTCTCACCACAGTATC 3'<br>3' AAACTAAGACACCTCCCCAT 5'
Tg-SNCA-F<br>Tg-SNCA-R<br>(Transgene: 469 bp<br>WT: No-bands)	5′-CAG GTA CCG ACA GTT GTG TAA AGG AAT-3′<br>5′-GAT AGC TAT AAG GCT TCA GGT TCG TAG TCT-3′

Note: Confirmation of plasmid transformation was done using primers provided in the kit by OriGene technologies and plasmid transfection in SH-SY5Y cells was confirmed using primers stated above.
